# Multitype Network-Guided Target Controllability in Phenotypically Characterized Osteosarcoma: Role of Tumor Microenvironment

**DOI:** 10.3389/fimmu.2017.00918

**Published:** 2017-07-31

**Authors:** Ankush Sharma, Caterina Cinti, Enrico Capobianco

**Affiliations:** ^1^Experimental Oncology Unit, UOS – Institute of Clinical Physiology, CNR, Siena, Italy; ^2^Center for Computational Science, University of Miami, Miami, FL, United States; ^3^Miller School of Medicine, University of Miami, Miami, FL, United States

**Keywords:** osteosarcoma cell lines, multitype networks, target controllability, protein network tomography, tumor microenvironment

## Abstract

This study highlights the relevance of network-guided controllability analysis as a precision oncology tool. Target controllability through networks is potentially relevant to cancer research for the identification of therapeutic targets. With reference to a recent study on multiple phenotypes from 22 osteosarcoma (OS) cell lines characterized both *in vitro* and *in vivo*, we found that a variety of critical proteins in OS regulation circuits were in part phenotype specific and in part shared. To generalize our inference approach and match cancer phenotypic heterogeneity, we employed multitype networks and identified targets in correspondence with protein sub-complexes. Therefore, we established the relevance for diagnostic and therapeutic purposes of inspecting interactive targets, namely those enriched by significant connectivity patterns in protein sub-complexes. Emerging targets appeared with reference to the OS microenvironment, and relatively to small leucine-rich proteoglycan members and D-type cyclins, among other collagen, laminin, and keratin proteins. These described were evidences shared across all phenotypes; instead, specific evidences were provided by critical proteins including IGFBP7 and PDGFRA in the invasive phenotype, and FGFR3 and THBS1 in the colony forming phenotype.

## Introduction

In biological networks, control theory addresses questions such as (a) how we decompose the structure of a complex network into components to simplify their functional interpretability? (b) Can redundant nodes and links be reduced to guarantee better network performance? (c) What are the effects of disrupting network connectivity by acting over particular nodes?

It would be useful to find a so-called network skeleton or core serving efficiently general inference purposes, possibly with no loss of information. Such skeleton is expected to be significantly smaller than the network, while reproducing its characteristic properties. However, what is *a priori* the most informative or essential or reproducible sub-network? In most cases, the answer is empirical. As a result, when the network structure changes one can measure the effects by monitoring what can be identified as critical hotspots. In an attempt to select subsets of nodes and links, controllability may involve the search of a minimum dominating set (MDS) (1). Being a minimal set not unique, this defines an NP-hard problem. Still, sets of the same size may differentiate by various node functional states, thus triggering a variety of connectivity paths and regulatory circuits.

Extending the application of such concepts to cancer networks is very tempting. Here, an assessment of controllability of influential nodes would be crucial to ensure that network integrity is sought against failures and attacks (2, 3). Key aspects in cancer are both monitoring the disease progression and evaluating the effects of therapies. However, exerting an effective control is complicated by the presence of a multitude of factors responsible of altering the normal physiological dynamics. When the latter are translated into gene or protein network dynamics, we would be interested in knowing what may change due to the insurgence of disease-related conditions. In general, two consequences may be observed: (a) intra-network state transitions, depending, for instance, on mutations affecting disease progression and (b) differential network configurations, elucidating the variations in connectivity patterns induced, for instance, by treatment effects.

Notably, a protein MDS was found enriched in disease, involved in regulatory functions and connected to protein complexes, thus legitimating a functional characterization in protein–protein interaction (PPI) networks (4). An existing categorization distinguishes between critical nodes (present in every minimal configuration), redundant nodes (never appearing in minimal sets), and intermittent nodes (appearing or not in minimal sets). Another recent study on large-scale PPI networks has classified proteins leading to disease mutations, viruses, and drug targets identification (5). Also, functional controllability was explored in epigenetically treated osteosarcoma (OS) cancer interactomes, and a module of sentinel nodes was identified as highly enriched in cancer hallmarks and marginally overlapping with both differentially expressed and mutated genes (6).

Here, we have considered experimental data susceptible of systems analysis. Specifically, the choice of OS is relevant from multiple viewpoints. First, it is a prevalent form of bone cancer with a relatively high incidence (second highest, overall) in young populations. In particular, metastatic OS shows less chances of survival (up to 30%).^1^ Second, from a genomic perspective, genome-wide OS studies have reported correlation between diffuse dysregulated gene expression with genomic aberrations (7). Third, focused cancer research has been provided for this cancer, delivering a wealth of knowledge in support of clinical studies (see EuroBoNet^2^) (8). These collections of OS cell lines and xenografts have been analyzed at both genomic and epigenomic levels (9–12). Of even greater interest to our study, further extended phenotypic characterization results have been proposed by a study centered on 22 OS cell lines (13). Among the OS phenotypic features that were examined, there were *in vivo* tumorigenicity (Tp or tumorigenic phenoptype) and *in vitro* colony-forming ability (Cp or colony-forming phenotype), together with invasiveness (Ip or invasive phenotype) and proliferation capacity (Pp or proliferation phenotype).

**Figure 1:**
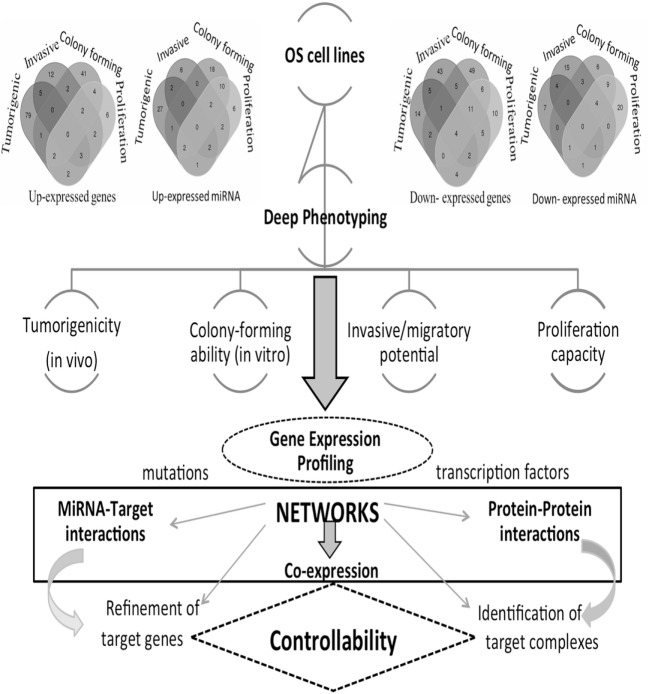
Computational and analytical flowchart. Differentially expressed gene (DEG) profiles are reproduced from each osteosarcoma (OS) cell line and comprehensive comparative analyses are derived. Venn diagrams show DEGs and DE miRNAs for the different phenotypes here considered: tumorigenic, invasive, colony forming, and proliferation. Different types of networks are employed: gene co-expression, miRNA-target, and protein–protein interaction networks, including drugs. These are then functionally annotated, including pathways and protein complexes. Deciphering cancer regulation networks suggests the application of control concepts. These are hard to implement, but this challenge may be transformed into a sequence of tasks solved with the help of accurately selected fractions of nodes and corresponding links describing critical features. This goal corresponds to setting a target control problem, whose solution requires the search for a minimum number of driver nodes. In real cancer networks, it is natural to expect that only approximate solutions may hold. Through the identification of targets in cancer networks, we can establish the cancer relevance of functional controllability.

These phenotypes reflect the OS heterogeneity that we here investigate through a network inference approach. In particular, a multitype network approach seems the most appropriate to deal with phenotypic characteristics underlying various transcriptional states and transcriptome–interactome regulation circuits involving various bioentities. The understanding of the regulation mechanisms is expected to drive the identification of novel OS therapeutic targets. However, there are currently no consistent results addressing the use and impact of networks for the identification of cancer targets. We propose, therefore, a novel direction, and Figure 1 provides the main steps of our integrative inference approach.

## Materials and Methods

### Controllability

Controllability of non-linear systems can be structurally approximated by canonical linear, time-invariant dynamics (14). Formally, the following representation holds: dx(*t*)/dt = Ax(*t*) + Bu(*t*), with *x*(*t*) = [*x*_1_(*t*),……, *x_N_*(*t*)] capturing the state of a network of *N* nodes at time *t*; *u*(*t*) an input vector of dim ension similar to A; A (*N* × *N*) describing system wiring by interaction strength between components; B (*N* × *M*, with *M* ≤ *N*) identifying node controllability due to external controller. Such system is controllable if can be driven from any initial state to any desired final state in a finite time. A controllability matrix C (Kalman Matrix) is an (*N* × *NM*) constant matrix that depends on system parameters and is defined as C = [B, A, A^2^B,…, A*^N^*^-1^ B]. Theory, following (15), says that a dynamical system is controllable if and only if it follows the Kalman’s controllability rank condition, i.e., Rank (C) = *N*.

### Spectral Decomposition

Controllability is associated with Spectral decomposition, another popular research direction in networks (see the following link for a few introductory concepts and a list of general references^3^). The primary aspect is that the steady-state configuration of a system or a network is proportional to its principal eigenvector (corresponding to the largest eigenvalue). In general, network eigenvalues are denoted by λ_i_ computed from the adjacency matrix, i.e., A *e*(λ) = λ *e*(λ), and ordered from 1 to *n* in descending order, such that λ_MAX_ = λ_1_ ≥ …. ≥ λ*_n_* forms a complete orthonormal basis.

In particular, it is important to check whether λ(1) corresponds to a localized state or to a delocalized state, which tells how the energy is distributed among spectral components. Notably, the modularity of the network is linked to such spectrum, and a property called participation ratio (PR) allows the quantification of the effective number of nodes significantly participating in a given eigenvector. When a concentration of such property occurs in just a few nodes, localization is observed. PR can be computed from the normalized eigenvector, as $e_{i}^{N}=e_{i}/e_{i}^{*}$, with the principal eigenvector as the denominator. Under normalization to unit in the L_2_-norm, it holds: $\text{PR}={\left[\sum_{i=1,n}e_{i}^{4}\right]}^{-1}$.

For the scopes of this work, it is of great relevance to compute the inverse participation ratio (IPR) (16, 17). This measure offers two limiting cases worth high consideration in target control situations. A value of 1/*n* indicates that the components are identically weighted, while a value of 1 indicates only one component is unitary and the rest as zero. In other terms, IPR indicates the reciprocal of the number of significantly contributing eigenvectors components. With regard to localization, in the limit of *n → inf*, IPR is O(1) (or tends to 1), and thus the eigenvector is localized (possibly at few nodes), *vice versa* the eigenvector is delocalized if IPR → 0.

Spectral techniques may identify specific proteins relevant for structural and functional network properties [see (18) for protein network tomography, or also (19) for related aspects]. Extremal eigenvalues are related to dynamical properties of the networks (20, 21). The largest eigenvalue in all phenotypes lies below 2 and the largest eigenvalue observed for Tp network shows the highest variance, playing an important role in linear stability and synchronization (22). The eigenvalue plots are useful to show the best fit for scale-free networks, and such evidence is observed in all four phenotypes, indicating that a few of their vertices are structurally dominant (Figure S5 in Supplementary Material).

### Cell Lines, Profiling, and Mutations

The examined OS cell lines are publicly available from GSE28425 (13). Also, 19 of 22 different OS cell lines were obtained from the resource EuroboNet. Recomputed differentially expressed genes (DEGs) could be grouped according to the characteristics of the cell lines (listed in Supplementary Material, Table 1). Data preprocessing from mRNA expression profiles was performed using the *Gene Expression* module v3.1.7 of *Illumina Bead studio* (v3.1.0.0). The *LUMI* package (R statistical framework) was used for variance stabilizing transformation and quantile normalization at the probe level. Intensity values were log-transformed and quantile-normalized for miRNA expression data. The fold change (FC) of the preprocessed microarray data, defined as ratio of the intensities between two groups of cell lines classified into different phenotypes (see Table 1), was log-transformed and computed with an empirical Bayes method from the packages *LIMMA* and *GEO2R* in *Bioconductor*^4^ (24). The adjusted *p*-value from the *T* test was then determined; and for multiple test correction, the false discovery rate method (FDR) was used (25). A cutoff of 1.5 was used for selecting DEGs, i.e., log_2_(FC) ≥1.5 or ≤−1.5. The variations and missense mutations for DEGs in each phenotype of the OS cell lines were retrieved from the cancer Gene census (26), from exome sequencing data of patient diagnosed with OS (27), and using three OS cell lines (28). All mutation types included in cancer gene census were missense, coding silent, and of unknown phenotype; when confirmed somatic, they were layered on the DEGs in each OS phenotype. DEGs were then used for network reconstructions, each associated with the specifically identified phenotype.

**Table 1:** Top-five differentially expressed genes (DEGs) (Top) and DE miRNAs (Bottom) in osteosarcoma (OS) phenotypes (C-I-P-T).

Reference phenotype	Gene symbol	log[fold change (FC)]	Shared phenotypes	Gene symbol	Log(FC)	Shared phenotypes
Tumorigenic Vs non-tumorigenic	*BGN*	3.492	I-T	*IL1A*	−2.221	P-T
	*MGP*	3.459	T	*EPB41L3*	−2.338	P-T
	*DKK1*	3.034	T	*NPPB*	−2.693	C-I-P-T
	*LOX*	2.873	T	*KRT17*	−2.752	C-I-P-T
	*TM4SF1*	2.74	T	*QPCT*	−3.081	I-T

Invasive Vs non-invasive	*DCN*	4.197	I-P-T	*KRT17*	−2.945	C-I-P-T
	*COL1A2*	2.963	C-I-P	*OCIAD2*	−2.98	I-T
	*S100A4*	2.775	I	*IGFBP7*	−3.213	I
	*S100A4*	2.602	I	*COL4A1*	−3.37	C-I-P
	*PDGFRA*	2.375	I	*IER3*	−3.959	C-I-P

Colony forming Vs non-colony forming	*COL1A2*	2.895	C-I-P	*C9orf58*	−2.963	I-P
	*HAPLN1*	2.852	C-P	*LAMA5*	−3.015	C-I-P
	*ALPL*	2.832	C	*COL4A1*	−3.126	C-I-P
	*KYNU*	2.572	C-I-P	*ACTG2*	−3.384	C-I-P-T
	*MAFB*	2.431	C-P	*NPPB*	−3.389	C-I-P-T

Proliferation Vs non-proliferation	*COL1A2*	2.804	C-I-P	*KRT17*	−2.606	C-I-P-T
	*MAFB*	2.544	C-P	*COL4A1*	−2.643	C-I-P
	*NDRG1*	2.316	P-T	*LAMA5*	−2.962	C-I-P
	*SNTB1*	2.009	P	*ACTG2*	−2.982	C-I-P-T
	*SPOCK*	1.979	I-P-T	*NPPB*	−3.046	C-I-P-T

**Reference phenotype**	**miRNA symbol**	**Log(FC)**	**Shared phenotypes**	**miRNA symbol**	**Log(FC)**	**Shared phenotypes**

Tumorigenic Vs non-tumorigenic	*hsa-miR-199b-5p*	5.6	P-T	*hsa-miR-133b*	−2.1	T
	hsa-miR-100*	3.66	I-T	*hsa-miR-449a*	−2.15	C-I-T
	*hsa-miR-222*	3.6	T	hsa-miR-181a-2*	−2.38	T
	*hsa-miR-136*	3.34	T	*hsa-miR-142-3p*	−2.73	T
	*hsa-miR-337-5p*	3.06	T	*hsa-miR-15a*	−3.9	T

Invasive Vs non-invasive	*hsa-miR-193a-3p*	2.94	I	*hsa-miR-598*	−3.2	I
	hsa-miR-100*	2.44	I-T	*hsa-miR-363*	−3.44	I
	*hsa-miR-99a*	2.41	I	*hsa-miR-34a*	−3.75	I
	*hsa-miR-193a-5p*	2.4	I	*hsa-miR-146a*	−4.29	C-I-P
	*hsa-miR-449a*	2.09	C-I-T	*hsa-miR-135b*	−5.7	I

Colony forming Vs non-colony forming	*hsa-miR-449a*	2.97	C-I-T	*hsa-miR-376c*	−2.67	C-I-P
	*hsa-miR-545*	2.77	C	*hsa-miR-146a*	−2.76	C-I-P
	hsa-miR-505*	2.57	C	*hsa-miR-497*	−2.82	C
	*hsa-miR-452*	2.47	C-P	*hsa-miR-124*	−2.91	C
	*hsa-miR-7*	2.45	C	*hsa-miR-155*	−5.89	C

Proliferation Vs non-proliferation	*hsa-miR-199b-5p*	3.28	P-T	*hsa-miR-146a*	−3.49	C-I-P
	*hsa-miR-452*	2.67	C-P	*hsa-miR-377*	−3.51	P
	*hsa-miR-34c-5p*	2.63	P	*hsa-miR-155*	−3.67	P
	*hsa-miR-152*	2.34	P	*hsa-miR-376a*	−3.69	C-P
	*hsa-miR-886-3p*	2.25	P	*hsa-miR-376c*	−3.94	P

*DEG profiles. (a) *Tp state*: BGN, encoding a member of the small leucine-rich proteoglycan (SLRP) family of proteins, related to bone growth, muscle development and regeneration, and collagen fibril assembly in multiple tissues, and regulating inflammation and innate immunity; MGP, inhibiting bone formation; DKK1, whose overexpression is associated with osteolytic bone lesions; LOX, encoding a member of the lysyl oxidase family of proteins with a role in tumor suppression, and crosslink collagen fibers in extracellular matrix (ECM), revealing a pre-metastatic niche in bones; TM4SF1, whose encoded protein is member of the tetraspanin family playing a role in the regulation of cell development, activation, growth, and motility; IL1A, an interleukin-1 cytokine involved in various immune responses, inflammatory processes, and hematopoiesis; EPB41L3, involved in multiple cancers. (b) *Ip state*: DCN, encoding a member of SLRP, mediating tumor suppression, autophagy, inflammation, and angiogenesis; COL1A2, encoding the pro-alpha 2 chain of type I collagen found in most connective tissues; S100A4, part of S100 proteins involved in the regulation of cell cycle progression and differentiation, and implicated in metastasis; PDGFRA, encoding a cell surface tyrosine kinase receptor for the platelet-derived growth factor family, with a possible role in tumor progression. (c) *Cp state*: ALPL, encoding a member of the alkaline phosphatase family of proteins, possibly linked to skeletal defects; LAMA5, part of Laminins, a family of ECM glycoproteins major non-collagenous constituent of basement membranes, and implicated in cell adhesion, differentiation, migration, and metastasis; ACTG2, involved in cell motility and cytoskeleton maintenance. (d) *Pp state*: NDRG1, member of the N-MYC downregulated gene family involved in stress responses, hormone responses, cell growth, and differentiation, whose encoded protein is necessary for p53-mediated caspase activation and apoptosis. *DE miRNA profiles*. *hsa-miR-146a*, which regulates inflammation and other innate immune system processes, is DE across phenotypes and is known to control cytokine signaling and toll-like receptors by binding to IL1 receptor associated kinase 1 (IRAK1); *hsa-mir-199b-5p* is highly upregulated in Tp and Pp. Also, these two phenotypes share the *DE hsa-miR-100* located in chromosome 11, which contains cancer susceptibility loci and is associated with multiple cancers; *hsa-miR-449a*, which exerts influence post-transcriptionally in various cancers, presents opposite regulation sign, and recent OS studies showed that when down-expressed, it suppresses tumorigenicity (*in vivo*) and promotes cell apoptosis (*in vitro*) (23)*.

### Co-Expression Networks

The *Weighted Gene Co-expression Network Analysis* (WGCNA) package (29) was used to reconstruct weighted gene co-expression networks for the DEGs by OS phenotypes and compared with normal bone samples. The scale-free property (most nodes are weakly connected and dominated by a few highly connected hubs) for networks was preserved using optimal β parameter during network reconstruction (Figure S1 in Supplementary Material). WGCNA computes edge weights on any two connected genes on the basis of the so-called topology overlap measure. Edge weights with values between 0 and 1 measure the expression correlation between connected genes and shared neighbor genes (cut-off edge weight 0.05). The networks were visualized using force directed graph drawing (Cytoscape v3.3). Centrality measures were computed using *Netanalyzer* and *Centiscape*. Hub and essential genes were calculated using degree distribution, betweenness centrality (BC), maximal clique centrality, and bottleneck nodes. Topological properties are described in the glossary (see Supplementary Material).

### Network Topology and Modularity

Centrality measures allow node or link ranking, and detection of intense traffic nodes and cross-linking network paths. Topological connectivity informs about the heterogeneity of networks (see Supplementary Material). Overlapping modules influencing community configurations were detected by *ModuLand via* local maxima search algorithms based on the Gradient Hill method (30). Modules were determined through an influence function calculated by *LinkLand* and *NodeLand*. The overall influence of the network is measured on each of its constituting nodes. Overlapping modules are identified on the basis of hills on the landscape, and each node of the network is assigned to the module with different strength.

### MicroRNA-Target and PPI Networks

miRNA-gene target interaction for DE miRNAs (Agilent microarray data) was extracted from *miRTarBase*^5^ (31) (this contains experimentally, validated miRNA-target interactions). The interactions data sources are 21 independent studies using reporter assays, western blots, and CLIP-Seq. We also extracted predictions from *Target Scan*^6^ (32). It searches 6- to 8-mer sites matching the miRNA seed region, with the support of an unbiased confidence score called context++ based on 14 features for miRNA targeting efficacy. A global human proteome interaction map was collected from public databases containing non-redundant, loops exempt, experimentally validated undirected physical protein–protein binary interactions. The extracted sub-networks for each phenotype consisted of known interactions of proteins (up to first order) for DEGs.

### Functional Annotations: GO, Pathways, Protein Complexes

GO annotations for DEGs were computed using *GEO2R*, using Bioconductor R packages for data analysis and transforms. The *BiNGO* plug-in was used for functional characterizations. Both FDR and Bonferroni corrections were used for multiple testing, the latter for molecular function annotation of the proteins containing variations. Note that pathway interaction cancer-specific data were retrieved from the Github repository^7^ (33) (recently integrated in NDEx, the Network Data Exchange database^8^) (34). The protein complexes were retrieved from the CORUM database^9^ (35), which manually annotated resources from mammalian organisms. Comprehensive annotations included protein complex functions, subunit composition, and cellular localization of complexes. Molecular functions are in Data S5 in Supplementary Material.

### Previous Evidence

Both mRNA and miRNA profiles have been identified in Ref. (13). For Tp, 354 significant DEGs were found, together with two DE miRNAs (*miR-199b-5p* and *miR-100-3p*). Further results were obtained for Cp, with 35 DEGs and one miRNA (*mir-155-5p*), for Ip, with 206 DEGS and two miRNA (*miR-135b-5p* and *miR-a46a-5p*), and for Pp, with 300 DEGs and 11 miRNAs. Functional enrichment from the cell line panel was also provided by the authors. Noticing that regulatory circuits are partly shared and partly distinctly characterizing OS phenotypes, it is natural to consider such complexity from a systems level viewpoint. Of interest also the fact that four genes—*COL1A2, KYNU, ACTG2*, and *NPPB*—were pervasively classified as DEGs. However, none of them in general is specific to OS. Special attention deserves *RUNX2*, a member of the RUNX family of transcription factors (known master regulators of development) encoding a nuclear protein with a Runt sequence-specific DNA-binding domain. The protein is essential for osteoblastic differentiation and skeletal morphogenesis, for which novel drug targets have been recently identified (36).

## Results

### Transcriptomic States: DEG and DE miRNA Profiling

**Figure 2:**
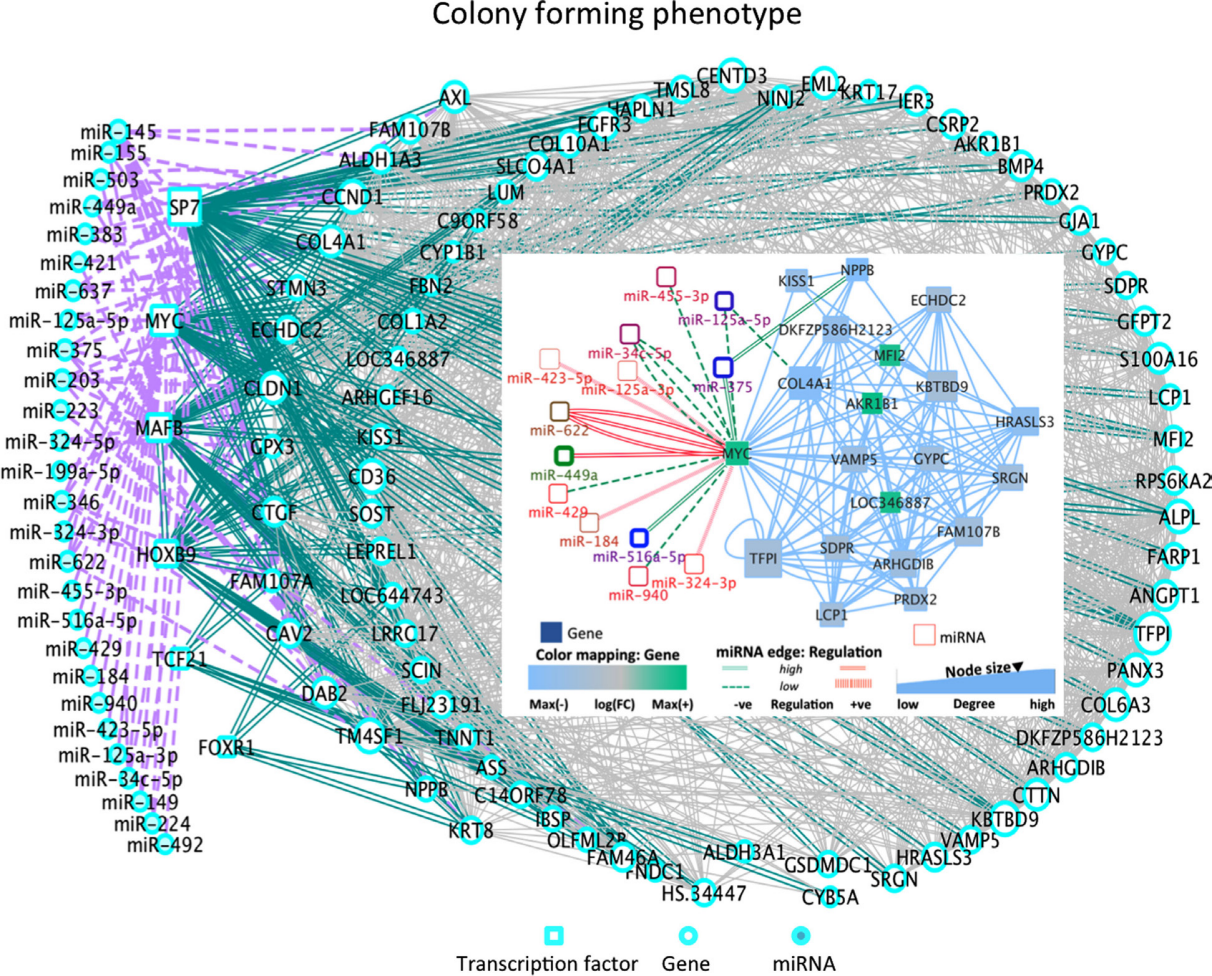
DE miRNA-TF co-regulatory dynamics in Cp (inset: C-MYC sub-network). *Cp state*: the overexpressed *hsa-miR-545* can induce cell apoptosis and cell cycle arrest by targeting CCND1 and CDK4. *Hsa-miR-7* is involved in major cancer pathways. The over-expressed *MYC* is involved with highly over-expressed *hsa-miR-449a* and *hsa-miR-622*, and with down-expressed *hsa-miR-516a-5p* and *hsa-miR-375*. Also, the over-expressed *hsa-miR-224* interacts with SP7 and *hsa-miR-199a-5p* interacts with MAFB [role in producing osteoblasts and osteoclasts, and in their differentiation]. The down-expressed *hsa-miR-492* interacts with TF Pod1 (TCF21), a tumor suppressor frequently silencing through epigenetic mechanisms. Other states present further aspects of interest (see Figure S3 in Supplementary Material): *Tp state*: the down-expressed *hsa-miR181a-2* shows deregulation in human cancers, and the down-expressed *hsa-mir181a* is pro-apoptotic and suppresses invasion and proliferation in OS (38). The over-expressed *miR142-3p* suppresses tumor growth, invasion, migration, and proliferation in OS cells. A hub appears between TFs and the DEG NFIX interacting with 50 partners, including DE *hsa-miR-375, hsa-miR-149, hsa-miR-324-5p* (down-expressed) and *hsa-miR-765, miR, hsa-miR-423-5p, hsa-miR149, hsa-mir361-5p* (over-expressed). Note *hsa-miR-375* also regulates hub DEGs (NPPB, PHLDA1, EMP1, and IGFBP) functional in cancer processes. *Ip state*: the down-expressed *has-mir-363*, suppressing invasion, migration, and OS cell growth through direct targeting of MAP2K4 (39) and the over-expressed *miR-193a* are correlated with PLAU, which modulates signaling in DNA damage, Notch, NF-κB, Myc/Max. *Pp state*: *Hsa-miR-152* is over-expressed here and in osteoblasts. Both *hsa-miR376c* and *hsa-miR-377* showed high down-expression, potentially suggesting a role in OS proliferation. Inverse correlation in *Hsa-miR-376c* and its target TGFA is observed in OS tissues and cell lines. Decrease in TGFA and its downstream signaling molecule’s expression due to over-expressed *mir-376c* is relevant in cellular proliferation and invasion in OS (40). Increased expression of *hsa-miR-377* with target CDK6 is already known to reduce cell proliferation and inhibit invasion in MG63 cell (41). No major TFs were DE in these cell lines.

Among the most altered genes in Table 1, ACTG2, NPPB, and KRT17 were significantly down-expressed in all phenotypes (Table 1; Data S1 in Supplementary Material). In particular, KISS1 is a gene suppressing melanoma (MEL) and breast cancer (BC) metastasis, and KRT17 shows up-expression that may be related to skin lesions and acts as a promoter of epithelial proliferation by regulating immune response. Tp, Ip, and Cp states shared molecular functions related to extracellular matrix (ECM) structural constituents containing collagen-related DEGs. Also the platelet-derived growth factor binding molecular function emerges. Phenotype-driven transcriptional states are summarized in Table 1 (with annotations). With regard to DE miRNAs, most are phenotype specific and very few miRNAs are shared (Table 1). Of interest also the convolution between the upregulated *hsa-miR-138* and MYC through target genes CDK2, CTNNB1, NFKB1, E2F4, and ITGA6 implicated in cellular processes related to focal adhesion, *NFKB*- and *RB1*-signaling (37) (Figure 2). MYC oncogene is overexpressed in >70% of human cancers and transcriptionally regulating cell cycle, cell death, senescence, cell adhesion, angiogenesis, genome stability, microenvironment, and metabolism.

### Interactomic States: Gene Co-Expression Networks

By using WCGNA, we reconstructed DEG-driven co-expression networks for all OS phenotypes. All co-expression networks appear in Figure S2 of the Supplementary Material. For instance, in Cp the high co-expression emphasizes functionally related gene sets. The network topologies reflect known properties, i.e., scale free and small world (see Figure S3 in Supplementary Material). Notably, redundant and diverse network configurations embed dynamics more difficult to control.

### miRNA-Target Gene Interaction Networks

All the miRNA-target gene networks appear in Figure S3 of Supplementary Materials. We reconstructed the miRNA-target gene interactions in each phenotype by only considering DE interactors. Tp and Cp present relatively higher clustering coefficient (see the glossary in Supplementary Material). This indicates that 1st degree node neighbors (dnn) tend to interact with each other (see Table 1 in Supplementary Material). Core skeletons (see glossary in Supplementary Material) in networks were formed by high DEGs in all phenotypes, showing high community centrality (CC) values (Data S2 in Supplementary Material). Tp genes included: FBXO32 (a muscle atrophy F-Box protein); EMP1 (epithelial membrane protein-1) with a role in cell–cell interaction and cell proliferation control; CDK4, a cyclin-dependent kinase important for G1 phase progression. Then, CCND1, which regulates CDK kinases, emerges in the other three phenotypes with very high CC (see Data S1 and S2 in Supplementary Material). Also, the top 10% genes with high degree, BC and CC showed gene regulation by miRNAs. The high DE tumor suppressor *miRNA-449-A* inhibits proliferation and prevents metastasis, and regulates the co-expressed GAS1 (putative tumor suppressor) and CDK4. Multiple lowly expressed miRNAs regulated genes with fewer interactions: *hsa-miR548b* and *hsa-mir342* interacted with DE hubs in the Tp miRNA-gene target network.

Note that *miR-342-3p* interacts with FBXO32, NDRG1, CAMK2N1, and RGS4, involved in cellular activation and communication, immune system, kinases, etc. The essential genes CCND1, CDK6, and GFRA1 formed the Ip core skeleton network sharing a multitude of miRNA interactions. The highly overexpressed *hsa-miR-182*, frequently amplified in MEL and experimentally known to promote metastasis and migratory potential, co-regulated the co-expressed CDK4 and CCND2, with the down-expressed GFRA1 and with the over-expressed NPTX1 and PDGFRA (involved in developmental cellular processes). In Cp state, high DE miRNAs such as *miRNA-449-A* also showed interaction with the hub connectors CCND1 and TXNIP (encoding a thioredoxin-binding protein member of the alpha arrestin protein family that regulates redox signaling, and possibly a tumor suppressor). Also, *hsa-miR-630* interacts with the DE CTHRC1, a known positive regulator of osteoblastic bone formation. The DEGs IGFBP5, CLDN1, and ALDH1A3 were found regulated by *hsa-mir-1224-5p*, along with other miRNAs such as *hsa-miR-603*, sharing interaction with hub genes CCND1, KYNU, and WISP1.

In Pp state, the top 10% essential connector genes (GFRA1, TXNIP, CCND1, and CCND2) of the core skeleton shared many miRNA interactions. CCND1 and TXNIP genes were regulated by the *miR520* family (*miR-520c-3p, miR-520d-3p, miR-520a-3p, miR-520e, miR-186*), which reduces secretion of pro-inflammatory cytokines by NF-κB signaling inhibition. The other regulator *miR-186* is known to suppress cellular proliferation, and *miR-423-5p* is known for autophagy regulation in cancer cells. The top DE miRNA, over-expressed *hsa-miR-449a, hsa-miR-542-3p, hsa-miR199a-3p*, and down-expressed *hsa-miR-338-3p, mir142-3p, miR28-5p*, have strong role in proliferation in multiple cancers, including OS *via* their target genes. Also, *hsa-miR-182* is known to interact with DEGs (NDRG1, NPTX2, CCND2, RRAGA, and GFRA1), targets in cellular proliferation.

### PPI Networks

Those associated with DEGs in each phenotype were extracted from non-redundant experimentally evidenced and curated sets of seed proteins in the human proteome. In Tp, proteins of the COL family (COL6A1, COL6A2, and COL6A3) appear, likewise Cathepsin (CTSB), interacting with PLAU and SLP1, and showing involvement in cellular processes related to collagen catabolic processes. In Cp, the majority of PPIs are involved also in cell migration and motility. In Ip, multicellular organismal development emerged. Finally, the biological processes involved in Pp interactions are related to ECM binding (complete annotations appear in Tables S2A–D in Supplementary Material). In each phenotype, the DEG-proteins showed few interactions and variation (Table S3 in Supplementary Material). As anticipated earlier, we also considered PPI networks expanded to their first order dnn (see Supplementary Material).

### PPI—miRNA Networks

The networks composed of interactions among DEG-related proteins and miRNA targets were reconstructed. The Tp state revealed limited heterogeneity, with a multitude of low DE miRNAs regulating proteins, namely the connector hub BCAS4 along with FBXO32, ADM, and CDK4 (Figure S4 in Supplementary Material). The down-expressed *hsa-mir-512-3p* regulated the over-expressed connector hub BCAS4, and NDRG1, a metastasis suppressor. The latter, along with ITGA11 and GAS1 proteins, plays a role in degradation of ECM and growth suppression and interacts with the highly DE *hsa-mir-449a*. Notably, the DE *hsa-miR-142-p* regulates SDC4, promoting LOX-dependent cross-linking of collagen, and providing bone health. The same miRNA then regulates IL1A, known to influence PLAU with regard to cancer invasion and metastasis. PLAU interacts with the highly over-expressed tumor suppressor *hsa-miR-193-3p*. Note that multiple miRNAs from the 14q32 locus associated with increased OS risk were DE wide interactors. Namely, FBXO32 interacts with the over-expressed *hsa-miR-431*, the down-expressed *hsa-miR-144* and *hsa-miR-377*, and other lowly expressed miRNAs from other loci. Then, *hsa-miR-494, hsa-miR-665*, and *hsa-miR-765* regulate PPP2R2B whose protein exerts negative control on cell growth and division. Also, its promoter methylation determines resistance to treatment with mTOR inhibitors. Finally, it contains missense mutations in OS patients. Another interaction is between *hsa-miR-144* and PHLDA1, which has missense mutations and whose protein shows anti-apoptotic effects of insulin-like growth factor-1.

**Figure 3:**
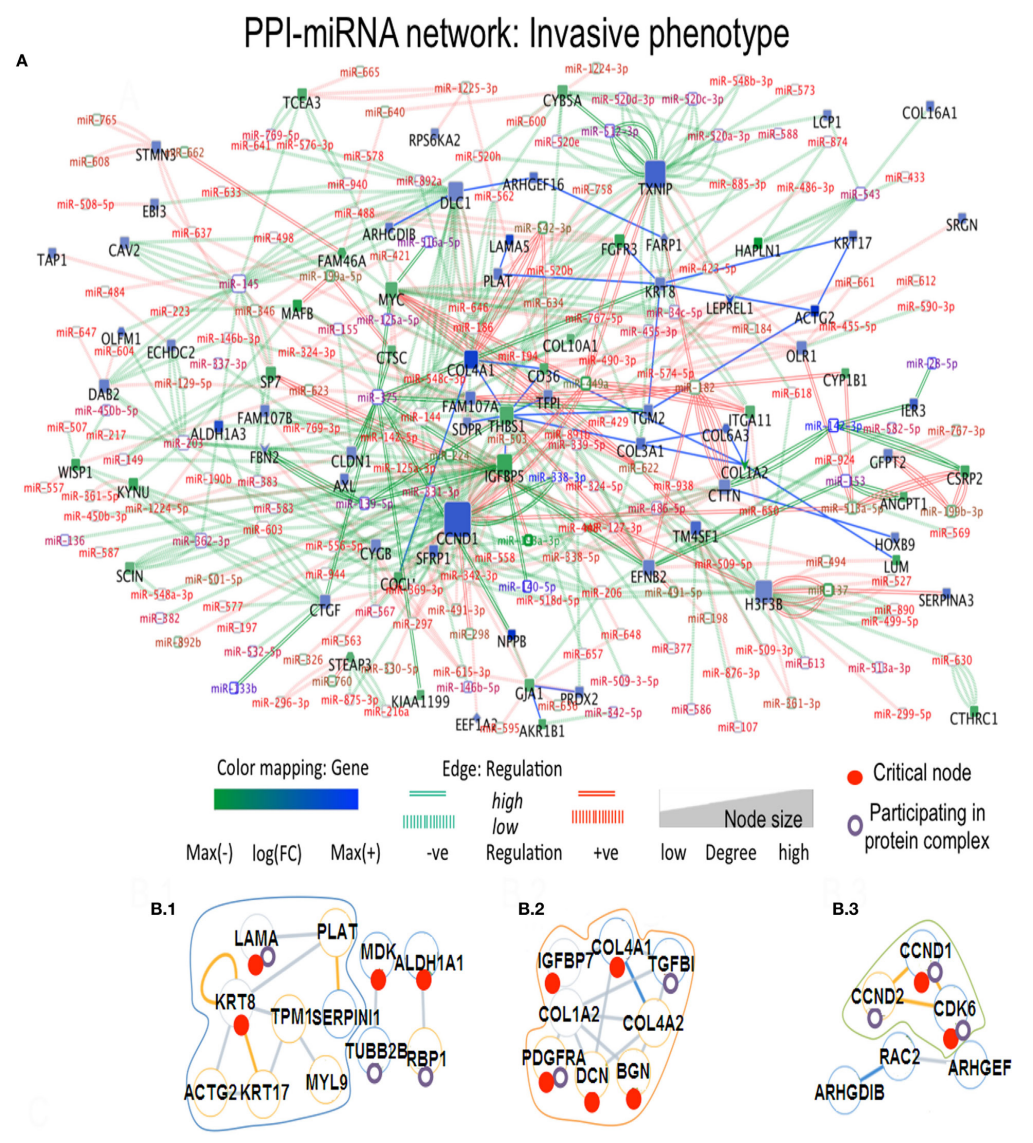

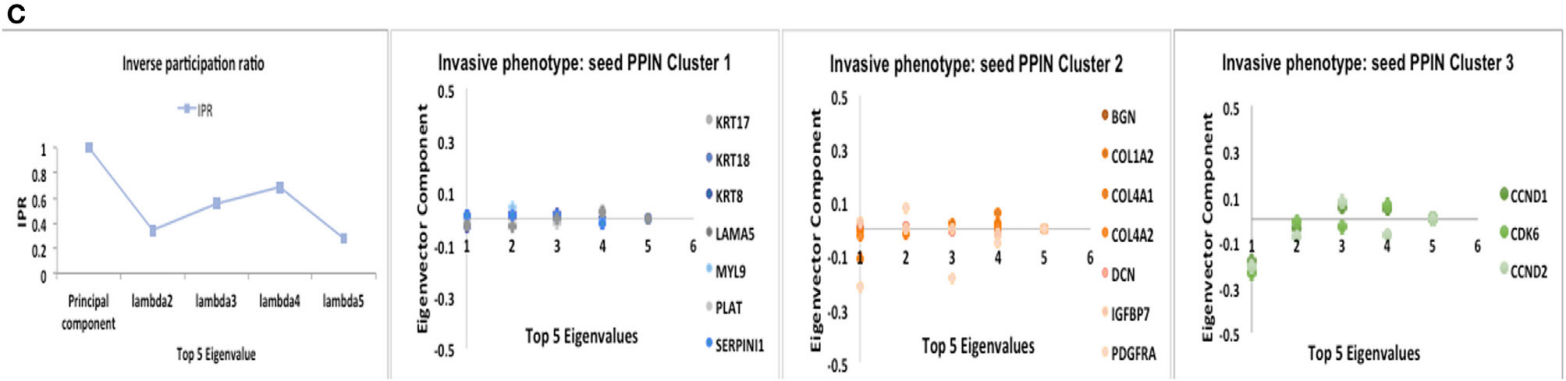
Composite targets in Ip. **(A)** Network configuration. **(B)** Identified sub-complexes. **(C)** TOP eigenvector component values corresponding to interacting seed proteins for the top-5 eigenvalues. The first eigenvector values refer to principal eigenvalues. Red dots denote critical nodes in first order PPIN networks and violet circles denote proteins participating in complexes. *Notes*: *miR-140-5p* regulates a critical node, ALDH1A1 and is classified as a critical link; critical nodes KRT8, COL4A2, COL4A1, and PLAT interact with other non-critical nodes.

**Figure 4:**
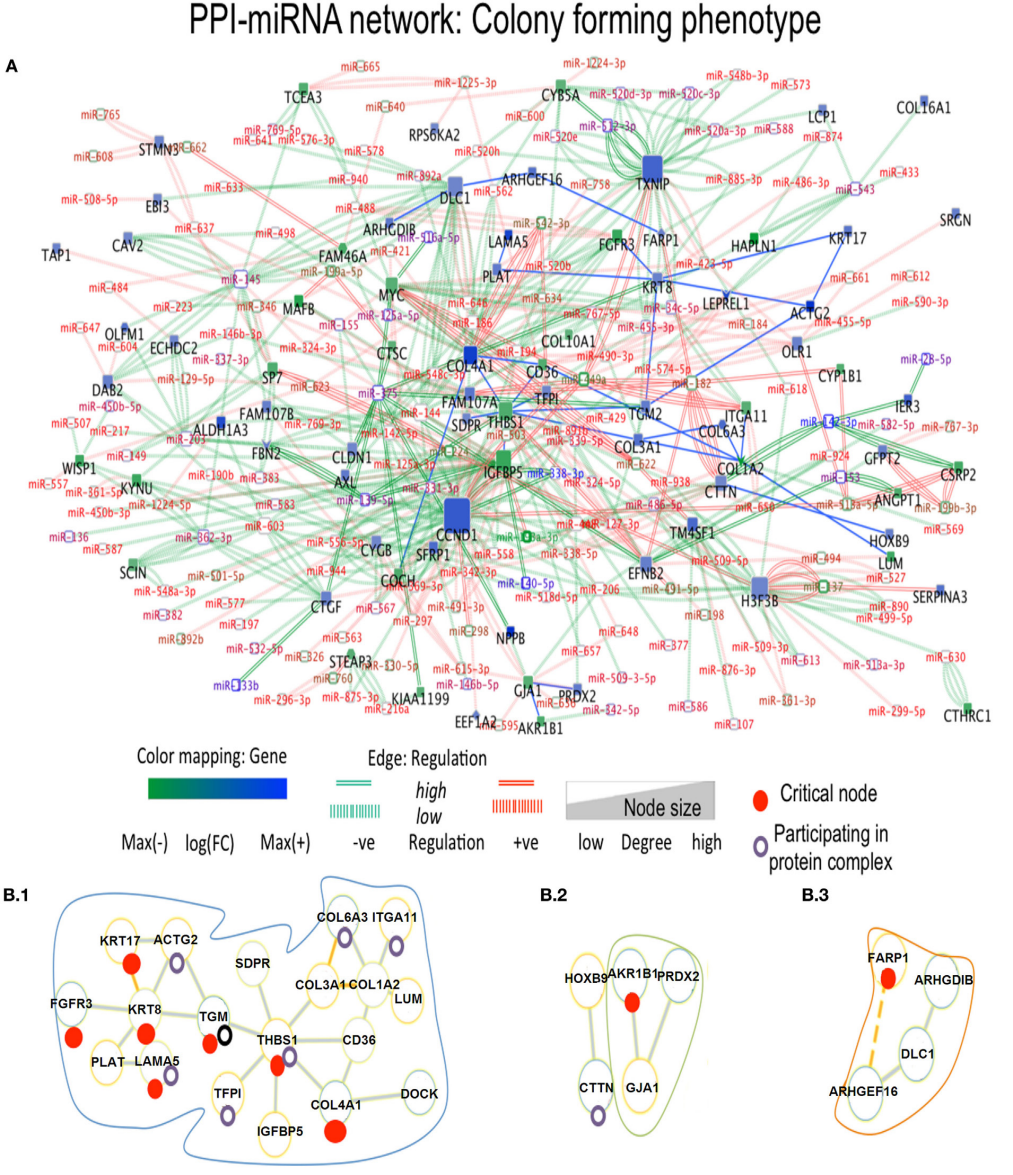

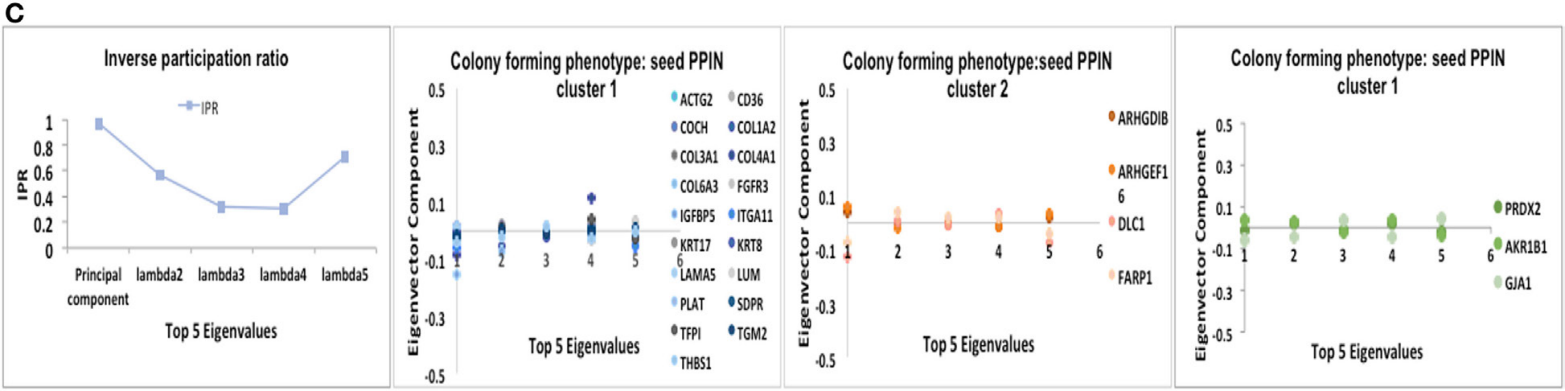
Composite targets in Cp. **(A)** Network configuration. **(B)** Identified sub-complexes. **(C)** TOP Eigenvector component values corresponding to interacting seed proteins for the top-5 eigenvalues. The first eigenvector values depend on principal eigenvalues. Red dots denote critical nodes in first order PPIN networks and violet circles denotes proteins participating in complexes. *Notes*: miRNAs constituted the majority of critical interactions along with critical nodes DLC1, ACTG2, and FARP1 showing interaction with other non-critical nodes, and critical node KRT8 interacts with another DE critical node ACTG2. FARP1 showed missense mutation and involvement in pathways related to RhoA regulation.

In Ip state, the connector hub protein PDGFRA is regulated by multiple miRNAs, including the over-expressed *hsa-miR-491-5p, hsa-miR-182, hsa-miR-298*, and the down-expressed *hsa-miR-140-5p* (42) (see Figure 3). The *hsa-mir-491* family is known to function in epithelial to mesenchymal transition and to influence cellular invasion and proliferation. The down-expressed *hsa-miR-298* interacts with the connector hub CCND1. COL4A1 and COL1A2 proteins, with unknown type mutations, are regulated by the DE *hsa-miR-767-5p*, showing functions related to oncogenic processes. *Hsa-miR-153* showed regulation of STMN2 protein having missense mutation (Table S3 in Supplementary Material). In Cp state, *hsa-miR-139-5p, hsa-miR144, hsa-miR217*, and *hsa-miR-615-3p* regulate the FBN1 protein containing missense mutation. The highly down-expressed *hsa miR-139-5p* shows anti-oncogenic and anti-metastatic effects, and is suggested to be a potent cancer biomarker (43). The down-expressed FARP1 protein (Figure 4) (critical node in PPI–miRNA network) interacts with the over-expressed *hsa-miR182*, which plays pivotal role in carcinogenesis. Importantly, FARP1 interacts with the lowly down-expressed *hsa-miR-874*, responsible for suppression of HDAC1 expression and enhanced Runx2 transcriptional activation during recovery of bone loss.

Finally, 17 DE miRNAs regulate thrombospondin 1 (THBS1), a connector hub in the Cp miRNA-PPI network, also regulated by *hsa-miR-139-5p* and *hsa-miR-144*, along with the highly over-expressed *hsa-miR-491-5P*, known to induce apoptosis and inhibition of AKT and MAPK, and leading to accumulation of the dephosphorylated BCL2L11 protein involved in anti- or pro-apoptotic regulation. Another interactor of THBS1 is COL4A1, a provincial hub interacting with numerous miRNAs and the high over-expressed *hsa-miR-542-5p*, promoting tumorigenesis and poor prognosis. In Pp state, provincial hubs appear (Figure S5 in Supplementary Material). CCND1 and CCND2 proteins interacting with MAFB show shared regulation by *hsa-miR-503*, a miRNA responsible for repression of cellular proliferation in fibroblasts (44). Multiple miRNA regulating each of these proteins were shared by also by GFRA1, the provincial hub TXNIP and then NDRG1. The highly over-expressed *has-mir-449a* and down-expressed *hsa-mir-512-3p* regulate TXNIP along with CCND1, GAS1, ITGA11, and NDRG1, and *hsa-mir-512-3p* increases the cellular proliferation and migration ability. The protein EEF1A1, containing missense mutation in OS patients, shows interaction with *hsa-miR-342-3p*, known to regulate variety of oncogenic processes, including cellular proliferation in different cancers. The OS phenotypes shared 32 cancer-related pathways (Table S4 in Supplementary Material) and comprised DEG-driven proteins either distinctly or jointly distributed.

### Effects of Controllability on Networks

**Figure 5:**
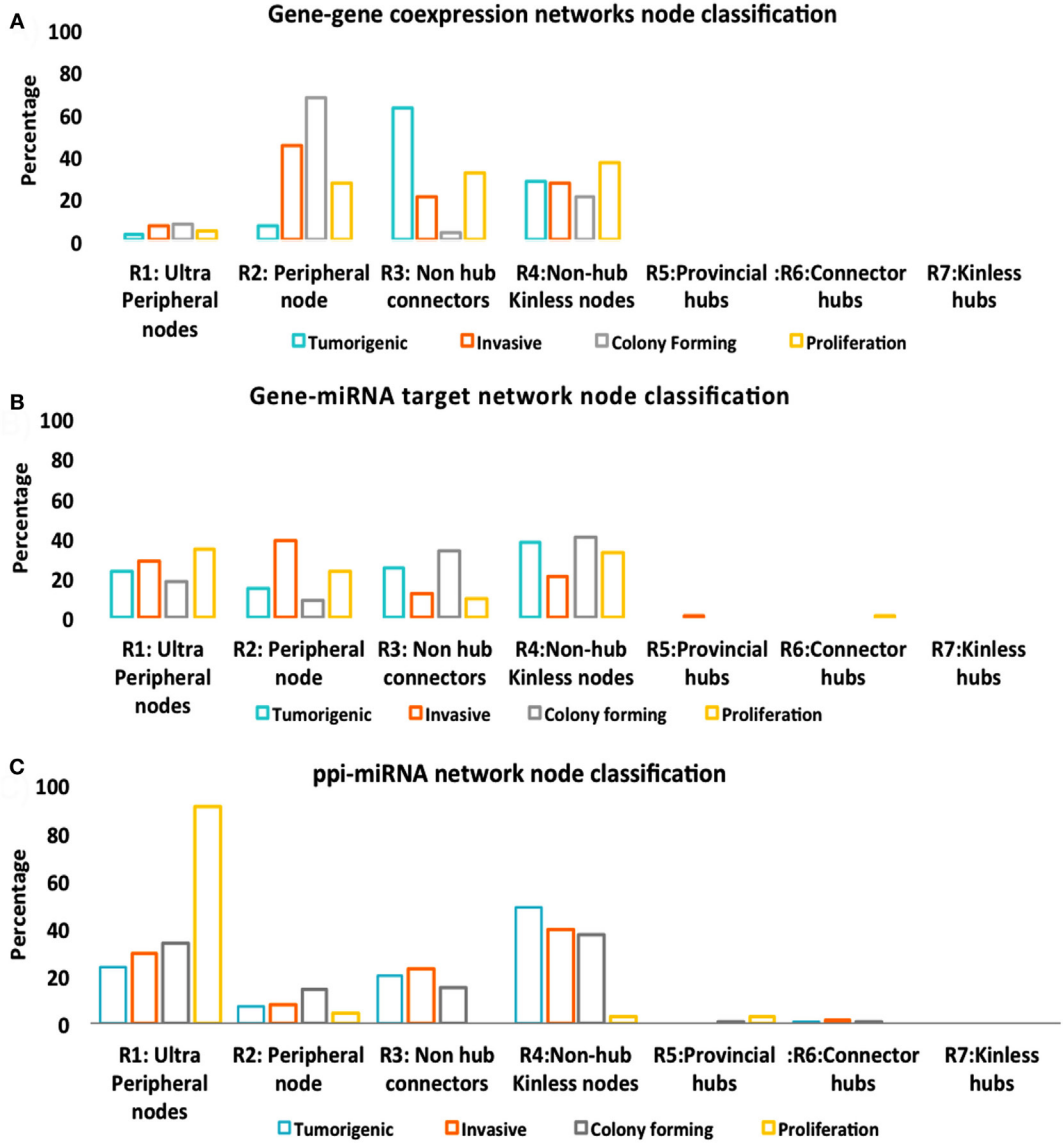
Node classification. **(A)** Gene–gene co-expression networks. **(B)** Gene–miRNA targets. **(C)** Protein–protein interaction (PPI)-miRNA target network.

**Figure 6:**
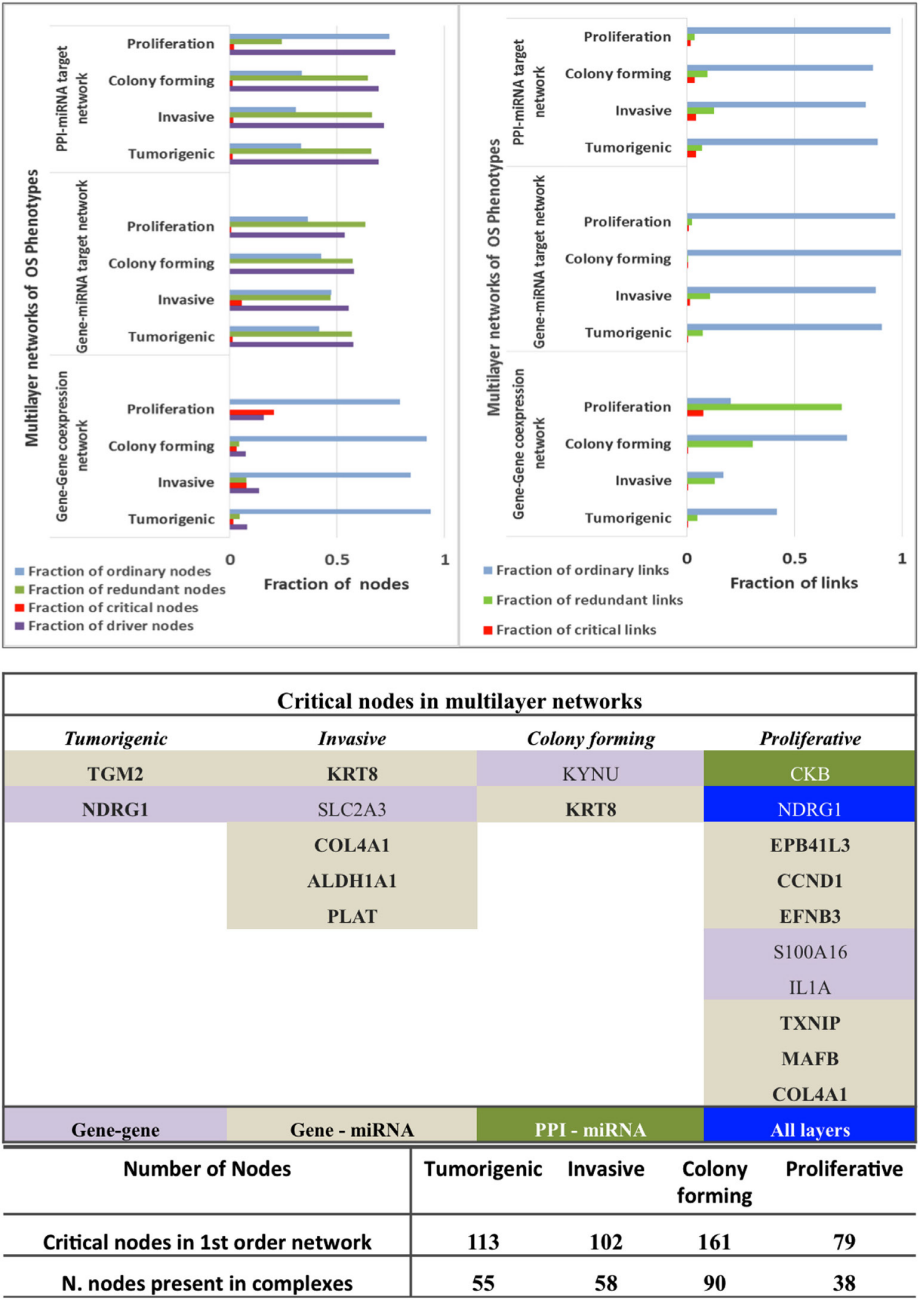
Controllability analysis. *Top panel*: gene–gene co-expression networks, miRNA-gene target networks and protein–protein interaction (PPI)-miRNA interaction networks showing occurrence of critical, ordinary, and redundant nodes. *Mid panel*: Critical nodes in multi-layered networks mapped to first order networks. *Bottom panel*: critical nodes computed in PPI first order networks and number of critical nodes in protein complexes that are manually curated and experimentally validated in CORUM database. Further statistics on classification of nodes in various networks is provided in Figure 5.

While Figure 5 described the classification of nodes in multitype networks, critical nodes have the highest presence in Pp state (Figure 6). With gene–gene co-expression networks, fewer critical nodes are in Tp and Cp states compared with Ip and Pp states. With gene–miRNA interaction networks, Pp state reveals many critical nodes, whereas Ip state contained none. Most miRNAs were classified as type 1 redundant nodes in all cell lines (Data S3 in Supplementary Material).

The critical nodes in multilayer OS networks were differentiated. Critical links in gene–gene co-expression networks revealed critical nodes in Tp state (FBXO32 and FLJ10154) and Ip state (OCIAD2, SLC2A3, COL1A2, NNMT, and GAS1), showing interaction with other non-critical nodes, whereas Cp state showed interaction among critical nodes WISP1 and TNNT1, especially. Pp state showed rich interactions among critical nodes, say NINJ2 that interacts with MAFB, CCND2, and with EPB41L3; then TMEM200A interacting with IL1A (interactions with non-critical nodes appear). With gene–miRNA interaction networks, Tp state showed critical links containing critical nodes interacting with non-critical nodes, whereas Ip and Cp states contained miRNAs in critical links. miRNAs *miR-183, mir155, miR-590-3p, miR-499-3p, miR-497*, and *miR-637* present critical interactions in Ip state that regulate important genes, similar to Cp state where *mir-630* regulates CTHRC1 and *miR149* interacts with C8orf55. In Pp state, critical links include critical node IL1A interaction with non-critical MAGEA10; then, the non-critical DE DCN interacts with FAM20C, and *miR-630* shows regulation relative to CTHRC1.

With PPI-miRNA networks, Ip and Cp states are showed in Figures 3 and 4, respectively. Instead, the Tp state contained miRNAs (*hsa-miR-186* and more) interacting with non-critical genes along with critical genes, such as FHL2, COL6A2, and NDRG1. In Pp state, only the critical node CKB showed interaction with kRT81 and the DE *miR-375* regulates highly DE NPPB genes (critical interactions). The miRNAs *miR-153, mir-342-3p*, and *miR-139-5p* regulated STMN2, EEF1A2, and DTX3, respectively. NDRG1, critical multilayer network node involved in stress responses, cell growth, differentiation, and metabolic pathway, is also critical for Tp and Pp states in first order PPIN. FBXO32 is critical in Tp gene–gene and gene–miRNA networks. Multilayer OS critical nodes, such as TGM (Tp state), KRT8 and COL4A1 (Ip state), KRT8 (Cp state), and CKB and COL4A1 (Pp state) (Figure 6) are also identified as critical nodes in corresponding PPIN first order networks (Data S3 in Supplementary Material), but with interactions lower than average degree. Redundant nodes in first-order PPIN across all phenotypes were peripheral. Serpin1, KRT18, and GAS1 (critical node in Ip state) are among the many hub nodes in different layers of biological networks and are regulated by a multitude of DE miRNAs.

### Protein Complexes

Critical nodes in multilayer networks participate in the selected protein complexes (Figure S5 and Data S4 in Supplementary Material). Notably, these refer to interactions with the tumor microenvironment (TME) of relevance for cancer progression toward metastasis. TME is known to contain distinct cell types, part of ECM-related macromolecules. We found that 48% of critical nodes in Tp and Pp PPI constituted complexes, while Ip and Cp ones reached 55.9 and 56.8% (Figure 6). Specifically, interacting critical nodes were identified in Ip protein sub-complexes: (i) LAMA5 encoding a laminin alpha chain (laminin is a family of ECM glycoproteins), implicated in cell adhesion, differentiation, migration, and metastasis; (ii) KRT8, member of the type II keratin family, and contributing to cellular structural integrity and cellular differentiation. (iii) DCN, encoding a protein of the small leucine-rich proteoglycan (SLRP) family (collagen fibril assembly) that binds to multiple cell surface receptors, influences tumor suppression by stimulating autophagy and inflammation and inhibiting angiogenesis and tumorigenesis (45–47); (iv) BGN, encoding a SLRP protein, also regulating inflammation and innate immunity; (v) COL4A1, a subunit of the type IV collagen playing a role in angiogenesis; (vi) IGFBP7, coding for an insulin growth factor binding protein (cell adhesion, cellular senescence, and autophagy); (vii) PDGFRA, encoding a cell surface tyrosine kinase receptor (tumor progression); and (viii) CCND1 (and CCND2), cell cycle regulatory proteins or D-type cyclins promoting cell cycle progression from G1 to S phase by binding to and activating the cyclin-dependent kinases CDK4 and CDK6. By aberrantly contributing to proliferation of cancer cells in a wide variety of human cancers, these kinases represent biomarkers and pharmacological targets in view of anticancer therapeutics (48, 49).

In the Cp network, distinct critical nodes are identified in (i) KRT17 (keratin), regulating epithelial cell growth (tissue repair) and stimulating Akt/mTOR pathway; (ii) FGFR3, encoding a member of the fibroblast growth factor receptor family, and interacting with fibroblast growth factors, and ultimately influencing mitogenesis and differentiation; (iii) THBS1, which encodes an adhesive glycoprotein that mediates cell-to-cell and cell-to-matrix interactions, active in platelet aggregation, angiogenesis, and tumorigenesis. The PPP2CA protein, a known tumor suppressor, is a pervasive critical node, also at first order PPIN level. The cAMP-dependent protein kinase catalytic subunit alpha complex containing critical nodes is shared between Tp, Cp, and Pp states, whereas the CD44 antigen-related complex is shared between Tp, Ip, and Pp states. Numerous proteins complexes containing critical nodes specifically characterize the Tp state: ERBB1 (EGFR), MMP14, and PLAUR; the Ip state: IKKB and RASA; the Cp State: GATAD2B, ACTB, ACTG1, NDUFA8, PPP2R2A, and SOS1; and the Pp state: CKB, RHOA, and AP2B1. In particular, GATAD2B and ACTB form the LARC complex. No interactions among proteins having missense mutations in OS were found in protein complexes.

### Eigen-Decomposition Results

The IPR measure (see Materials and Methods) for lowest non-zero eigenvalues in PPI-miRNA network were twofold higher for Tp (IPR = 1.7) as compared with Pp (IPR = 0.7). The IPR for the lowest non-zero eigenvalue in Ip and Cp networks was 1.4 and 1.19, respectively. The lowest non-zero eigenvalues that were observed for Pp network indicate presence of strong communities (i.e., nodes with fewer connections between groups than within groups and behaving nearly as disconnected components and resulting in non-zero eigenvalues). The eigenvectors are also associated with the lowest non-zero eigenvalues, still with higher IPR (Figure S5 in Supplementary Material).

The eigenvector scatterplots of Figure 3 with the five largest eigenvalues, and referred to the seed interacting proteins and critical nodes in Ip PPI complexes, showed variable bar length, i.e., eigenvector component values not concentrated in a single state but distributed among multiple energy states. Higher values appear for eigenvector component referred to the ALDH1A1 and the RBP1 proteins, interacting with DEG proteins (encircled in blue, C row) in the fifth largest eigenvalue χ_5_. The Tp and Pp plots for eigenvector components (Figure S4A in Supplementary Material; Figure 4B) demonstrate similar pattern for principal eigenvalue. The critical nodes identified in PPI-miRNA networks show extremal values (high negative or positive) for some of the interacting proteins in Tp; this appears in the eigenvector plots for lowest non-zero eigenvalues (Figure S5 in Supplementary Material). In Ip, the critical nodes CCND1, CCND2, and CDK6 participating in the B.3 complex showed eigenvector components approaching 0.2 for each node, suggesting delocalization. The other connected cluster containing critical node PDGFRA participating in the B.2 complex, along with critical node TGFBI, showed eigenvector component localized around zero. In Cp, the connected component contains critical nodes TGM2 and LAMA5 linked to another critical node THBS1 participating in many important complexes (Data S4 in Supplementary Material), and showing very localized eigenvector component. The connected component of seed proteins in Tp also includes the FHL family of proteins participating in complexes, and the critical protein TGM2, which interacts with ACTG2 while participating in different complexes. Pp contained only the LAMA5 protein involved in complexes.

Figures 3 and 4 refer to examples of protein complexes considered as possible candidate targets and retrieved from miRNA-PPIN configurations. The eigenvalues plotted with the IPR, which quantifies the number of states for a particle, and the eigenvector components (nodes, proteins) localization, or delocalization help to emphasize the target potential. High localization is equivalent to IPR telling that the distribution is concentrated on a few nodes/proteins. Lack of concentration indicates that a set of interacting proteins, participating to a sub-complex, may better identify a potential composite target. Specifically, pieces of evidence for a couple of phenotypes are proposed (other pieces of evidences are in Figure S4A in Supplementary Material; Figure 4B). The local context of a node in terms of interconnectivity patterns is relevant, therefore, to identify the potential of the candidate target beyond the individual node, thus identifying a composite target that can elucidate the functional relevance of the node itself based on the other interacting nodes. The presence of identified critical nodes in target sub-complexes brings additional value, as this means improved wide-spectrum controllability.

### Drug Interactions, Repositioning, and Repurposing

**Figure 7:**
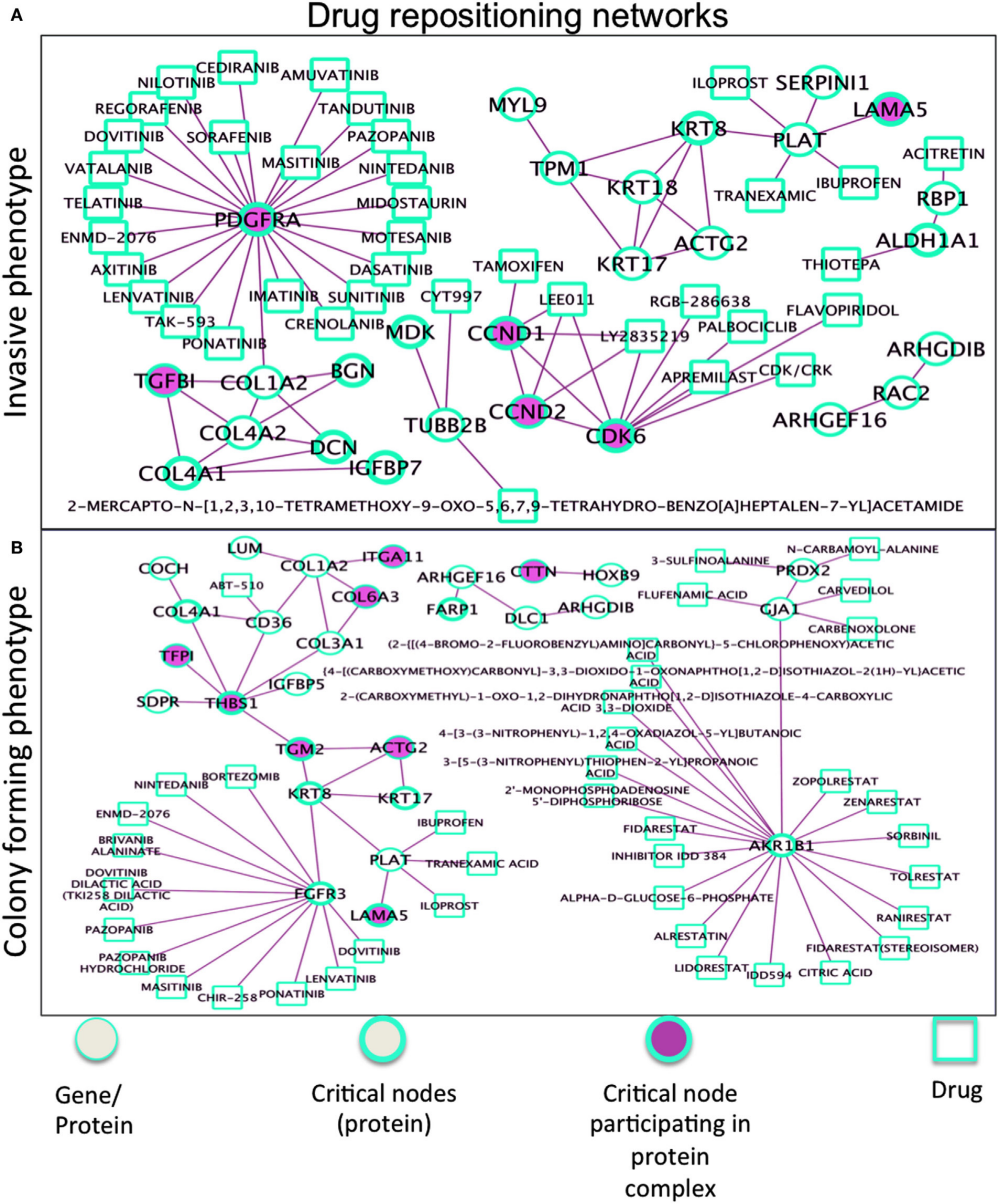
Drug repositioning networks for **(A)** Ip and **(B)** Cp. The R/Bioconductor package rDGIdb is used, as an R wrapper to query the drug–gene interaction database (DGIdb). As a result, PDGFRA has interactors, such as imatinib, dasatinib, sunitinib, sorafenib, pazopanib, and nilotinib, none specific and all inhibiting different kinases [i.e., imatinib also KIT and AB1 (with dasatinib used for imatinib resistance), sunitinib also VEGF and FLT3 (like crenolanib too), sorafenib also RAF etc], which might reveal advantageous. Two other networks in Figure S6 in Supplementary Material. In Tp, critical nodes CTSB and PLAU widely interact with drugs. MAOA interacts with antidepressant drugs, associated with decrease in bone mineral density and increasing risk of fracture. The Pp network proteins participating in complexes showed limited interactions with drugs, Pyridoxal Phosphate interacts with KYNU (collagen) relevant to osteosarcoma. The list of interactions is available in Data S6 in Supplementary Material.

Drug repositioning involves discovery of new roles for drugs, especially those with high failure rate and long-term development. Our phenotype-driven networks embedding critical nodes may gain further relevance when associated drugs are considered (Figure 7). The comprehensive resource here used is drug–gene interaction database (50, 51), with drug–gene interaction data from 15 different resources (52, 53). In the Ip drug-target network, the well-known Tamoxifen shows interaction with the CCND1 protein participating in complexes with CCND2 and CDK6 and also share interactions with other drugs, namely LEE011 (ribociclib), and LY2835219 (abemaciclib), both CDK4/6 inhibitors. Considering then the target PDGFRA (overexpressed in Ip), a drug compound of interest is lenvatinib (multiple kinase inhibitor), then regorafenib (multikinase inhibitor targeting angiogenesis, stromal/microenvironment and oncogenesis), and also nintedanib (small molecule tyrosine-kinase inhibitor, targeting VEGFR and FGFR). Inhibition of PDGF receptor signaling (with antibodies or DNA aptamers) has proven useful for treating cancer patients, leading to the development of different types of antagonists of its signaling, such as binders targeting the receptors and preventing their activation or promoting their degradation, and low molecular inhibitors of the receptor kinases. In the Cp network (bottom panel), AKR1B1 (member of the aldo/keto reductase superfamily, which consists of more than 40 known enzymes and proteins) showed interaction with many drugs, likewise FGFR3 (member of the fibroblast growth factor receptor family) emerged to be second reactive protein interacting with other cancer treatment drugs. Also, THBS1 appears (an adhesive glycoprotein mediating cell-to-cell and cell-to-matrix interactions, and involved in platelet aggregation, angiogenesis, and tumorigenesis), but with no drug interactors.

## Discussion

Despite inspiring much of the initial network literature, reverse engineering revealed limitations for dynamical biological systems. These need extended sensitivity tests for assessing parameter inferability (54). Two recent changes occurred: model systems have started to include enormous data volumes (big data), leading network inference approaches to unprecedented sophistication (multilayer networks). Generalizations such as reciprocal engineering (the interactome scaffold connecting pieces of experimental evidence and determining the target pathways), and forward engineering (pathway modulation used to analyze downstream phenotypes) (55). More importantly, controllability has emerged as a paradigmatic example of research direction with almost ubiquitous applications.

Multiple phenotypically differentiated OS cell lines may clarify target relationships. Our inference approach is centered on networks. One aim was to exert control on targets, single and composite ones, with the latter benchmarked to protein complexes. Pooling together heterogeneous evidenced data creates the premises for the analysis of systemic regulation dynamics of difficult replicability or interpretability. Deciphering such complexity requires multi-type networks. Because nodes and links represent genes, miRNAs, proteins, transcription factors, etc., the corresponding associative dynamics have relevance depending on their integrability. As a result, the identified OS targets were characterized by critical proteins, individually relevant or interacting in sub-complexes. Examples were offered by SLRP proteins and D-type cyclins, but distinct effects were also emerging from IGFBP7 and PDGFRA, critical proteins in invasive conditions, and from FGFR3 and THBS1, appearing in colony-forming phenotype. Collagen, laminin, and keratin proteins were shared across phenotypes.

It is clearly relevant the emergence of TME due to these identified targets. We stress the fact that the evidenced targets are connected, which suggests that multidrug targeted approaches may be particularly indicated. Such multiplicity of targets across OS phenotypes increases the overall complexity, but naturally reflects the role played by TME in this disease, and also justifies the ongoing phase I/II trials as important steps for more critical assessment of TME in OS pathogenesis (56).

We have then observed a few other specific aspects: (A) from the same reference system of pan-cancer cell lines, results depend on the computational tools used for the analysis. For instance, profiling the data discriminates among many measurements and their bio-annotations, all subjected to various degree of stringency to establish significance. But profiling is not sufficient, and calls for further inference shifting from the analysis of signatures of individual bioentities to the analysis of modules of connected bioentities; (B) shared and distinct features emerging at phenotype levels may vary quite substantially, while receiving influence from the adopted measurement system, and the best way to put forth causative instead of confounding effects is to evaluate pieces of evidence at a systems level and to exploit the embedded metrics to leverage their possible linkages; (C) networks are naturally differentiated, depending on data characteristics (OS phenotypes, in our study), but also on the object of investigation, targets in our case. Starting from the topological properties, we achieved accurate analyses through controllability and spectral concepts, so far widely unexplored, but with potential toward target discovery.

In dynamical systems, steady-state network configurations are usually considered to be proportional to the principal eigenvector corresponding to the largest eigenvalue. The residual eigenvectors refer to non-steady-state conditions, addressing system disequilibrium. Network modularity reflects the eigenvector properties, and allows measurement, for instance, through the PR, which quantifies the effective number of network nodes representing significant eigenvector components. In scale-free networks, such components tend to be localized in a few well-connected nodes. Correspondingly, the IPR indicates the reciprocal of the number of eigenvector components offering a significant contribution, thus measuring the localization degree of a particular eigenvector. A recent application of network controllability for a large-scale study aimed at identifying disease genes and drug targets (5). Differently classified nodes allowed to assess distinct functional and regulatory roles. Controllability pinpointed hotspots (“fragile nodes”) informative about state transitions from health to disease. Critical controllability was examined both structurally (PPIN) and functionally (transcriptome) in large-scale integrated systems, associating critical nodes and drug targets (57). We reconciled these characteristics by proposing novel strategies to identify a variety of targets within OS phenotypic heterogeneity. Especially, exerting control on composite targets might lead to improved drug repositioning or repurposing^10^ with cost-effectiveness advantages for cancer therapy.

## Ethics Statement

This study has not involved patients, being based on publicly available data from experimental studies on a panel of cell lines.

## Author Contributions

AS: performed method computations and data analyses; drafted manuscript parts. CC: reviewed the experimental evidences and the biological findings. EC: conceived and designed the methodological pipeline and wrote the manuscript. All authors approved the manuscript in its final form.

## Conflict of Interest Statement

The authors declare that the research was conducted in the absence of any commercial or financial relationships that could be construed as a potential conflict of interest.

## Footnotes

^1^http://www.cancer.org/cancer/osteosarcoma.

^2^http://eurobonet.pathobiology.eu/.

^3^https://en.wikipedia.org/wiki/Spectral_graph_theory.

^4^https://www.bioconductor.org/.

^5^http://mirtarbase.mbc.nctu.edu.tw/.

^6^http://www.targetscan.org/vert_71/.

^7^https://github.com/NCIP/pathway-interaction-database.

^8^http://www.ndexbio.org/.

^9^http://mips.helmholtz-muenchen.de/corum/.

^10^https://ncats.nih.gov/preclinical/repurpose.

## Supplementary Material

Supplementary Data S1 **Sheet 1:** Comparison of log (FC) of miRNAs analyzed by Namløs et al. (10) and log(FC) computed in each OS phenotype. *Sheet 2*: Differential expression of miRNAs in (A) tumorigenic, (B) invasive, (C) colony forming, and (D) proliferation. *Sheet 3*: Centrality statistics of gene–gene co-expression and miRNA-target genes. Hubs in both networks. *Sheet 4*: Various centrality measures of DEGs in Tp (tumorigenic Vs non-tumorigenic cell lines). *Sheet 5*: Various centrality measures of DEGs in Ip (invasive Vs non-invasive cell lines). *Sheet 6*: Various centrality measures of DEGs in Cp (colony forming Vs non-colony forming cell lines). *Sheet 7*: Various centrality measures of DEGs in Pp (proliferation Vs non-proliferating cell lines).

Supplementary Data S2 **Sheet 1:** Centrality statistics of gene–gene co-expression networks. Hubs in gene–gene co-expression networks. *Sheet 2*: Controllability status of nodes: 0 = critical, 1 = redundant, 2 = ordinary in type I and type II along with various centrality measures for nodes in tumorigenic DE miRNA-DE target gene interaction network. *Sheet 3*: Controllability status of nodes: 0 = critical, 1 = redundant, 2 = ordinary in type I and type II along with various centrality measures for nodes in invasive DE miRNA-DE target gene interaction network *Sheet 4*: Controllability status of nodes: 0 = critical, 1 = redundant, 2 = ordinary in type I and type II along with various centrality measures for nodes in colony forming DE miRNA-DE target gene interaction network. *Sheet 5*: Controllability status of nodes: 0 = critical, 1 = redundant, 2 = ordinary in type I and type II along with various centrality measures for nodes in proliferation DE miRNA-DE target gene interaction network.

Supplementary Data S3 **Sheet 1:** Classification type I nodes in multitype networks for all OS phenotypes. *Sheet 2*: Fraction of nodes and links in multitype networks for all OS phenotypes. *Sheet 3*: (A) Critical nodes computed in gene–gene co-expression, miRNA-target gene interaction, PPI-miRNA and PPI first order interaction networks. (B) Number of critical nodes in first order and number of critical nodes present in protein complexes from the CORUM database. *Sheet 4*: Information related to critical nodes identified in PPI first order network having differential expression in other OS phenotypes. *Sheet 5*: Classification of type I links in multitype networks for all OS phenotypes.

Supplementary Data S4 **Sheet 1:** Details of seed proteins corresponding to DE genes participating in experimentally determined protein complexes stored in CORUM database. *Sheet 2*: Details of proteins (gene symbol, Uniprot id) participation in complexes experimentally determined in different organisms (systems) stored in CORUM database. *Sheet 3*: Detailed information on the proteins present in protein complexes containing critical nodes shared and specific to OS phenotype-first order PPIN. *Sheet 4*: Information of complete list of complexes present in CORUM. Gray shaded cells contain complexes with involvement of c-MYC protein.

Supplementary Data S5 **Sheet 1:** Molecular functions of top-10 overlapping core proteins of identified modules of tumorigenic PPI first order network for DEGs in OS phenotypes. *Sheet 2*: Molecular functions of top-10 overlapping core proteins of identified modules of invasive PPI first order network for DEGs in OS phenotypes. *Sheet 3*: Molecular functions of top-10 overlapping core proteins of identified modules of colony forming phenotype PPI first order network for DEGs in OS phenotypes. *Sheet 4*: Molecular functions of top-10 overlapping core proteins of identified modules of proliferation phenotype PPI first order network for DEGs in OS phenotypes. *Sheet 5*: Details in protein-pathways interactions shared between and specific to OS phenotypes.

Supplementary Data S6 **Sheet 1:** Drug—gene interactions for DEGs retrieved from the dGIDB database using RdGIdb package for R with details on number of experimental verification for (A) tumorigenic, (B) invasive, (C) colony forming, (D) proliferation.
