# Supplementary material for: Multitype Network-Guided Target Controllability in Phenotypically Characterized Osteosarcoma: Role of Tumor Microenvironment
Source: Front Immunol. 2017 Jul 31;8:918. doi: 10.3389/fimmu.2017.00918 (PMC5536125; doi:10.3389/fimmu.2017.00918)
Supplement: Supplementary file 1 [file presentation_1.pdf]

Multitype Network-guided Target Controllability in Phenotypically Characterized Osteosarcoma: role of Tumor Microenvironment

SUPPLEMENTARY MATERIALS

Ankush Sharma,[1,2] Caterina Cinti[1] Enrico Capobianco[1,2,3]

[1] Experimental Oncology Unit, UOS - Institute of Clinical Physiology, CNR - Siena (IT)

[2] Center for Computational Science, University of Miami, Miami - FL (USA)

[3] Miller School of Medicine, University of Miami, Miami – FL

**Corresponding author: ecapobianco@med.miami.edu*.

Table of contents

Glossary: pg 2-3.

Supplementary text: pg 4-7.

Supplementary references: 7- 9

Supplementary Tables: pg 9-31.

Supplementary Figures: 32-38.

1: Glossary of network terms.

Network Adjacency Matrix. The adjacency matrix A of a labeled graph is a matrix with rows and columns labeled by graph vertices, with a 1 or 0 for (v_i ,v_j) according to whether v_i and v_j are adjacent or not (Weisstein, Wolfram Web Resource).

Node Degree Distribution. Measure that reflects the local connectivity of a node and indicates the number of connections with other nodes, and is the probability distribution of degrees over the whole network. Most real world networks exhibit the scale free property, and the degree distribution follows a power law.

$$D(i) = \sum_{j=1}^{N} a_{ij}$$

where a_{ij} is the generic element of A, and N is the total of nodes.

Clustering Coefficient. Is given by the proportions of links (interactions) between the neighbor nodes divided by the maximum possible connection with the neighborhood.

$$CC(i) = \frac{2e_i}{k_i(k_i - 1)}$$

where e_i is the number of edges between the neighbors of '*i*', and k_i is the number of neighbors.

Shortest Path length: The shortest possible path (distance) between two nodes in a network. The shortest paths between all pairs of nodes are identified using the Dijkstra's algorithm (Dijkstra, 1959).

Average path length: Average of hops along the shortest paths for all pairs of nodes. It calculates the measure of efficiency of information. Most real world networks exhibits very short average path length, from which the small world concept comes (Watts and Strogatz, 1998;Freeman, 1977). Given the unweighted graph **G** of vertices **V**, let **d (** $\boldsymbol{v_1}$,$\boldsymbol{v_2}$), (where $\boldsymbol{v_1 v_2 \epsilon V}$ denote the shortest distance between $\boldsymbol{v_1}$ *and* $\boldsymbol{v_2}$). Assume that **d (** $\boldsymbol{v_1}$,$\boldsymbol{v_2}$) = 0 if $\boldsymbol{v_1}$ = $\boldsymbol{v_2}$ or $\boldsymbol{v_2}$ cannot be reached from $\boldsymbol{v_1}$. Then, the average path length l_G is:

$$l_G = \frac{1}{n.(n-1)} . \sum_{i,j} d(v_i, v_j)$$

Where ***n*** is the number of vertices in ***G.***

Network Diameter: Longest shortest path length (maximum length of shortest paths) between two nodes in whole network. It can be simply detected after the calculation of shortest path lengths from every node to all other nodes: the longest of all calculated shortest path length is the diameter. Therefore, the network size.

Betweenness centrality: Global centrality measure computing the load placed on a given network node, or sum of the fraction of shortest paths between all pairs of nodes that traverse through node *i*. By (Freeman, 1977):

$$B(I) = \frac{1}{(N-1)(N-2)} \sum_{J \neq I \neq K} \frac{G_{JK}(I)}{G_{JK}}$$

where g_{jk} is the n. of shortest paths between nodes j and k, and g_{jk} (i) is the n. of these shortest paths traversing through node i.

Closeness Centrality: Global centrality metric used to determine critical nodes in networks and is defined as the inverse of farness, which in turn, is the sum of distances to all other nodes (Opsahl et al, 2010). It provides information on how fast information spreads from one particular node, say *"**S**",* to all other nodes sequentially. The closeness of node *i* is defined as inverse of average path length (shortest path) from node *i* to all other nodes in the network. The measure for the connected component is as follows:

$$C(i) = \frac{N-1}{\sum_{i \neq j} d_{ij}}$$

where $\mathbf{d_{ij}}$ is the shortest path from residue i to residue j, and N is the total number of nodes. (Guimera' et al, 2005) rewrote the equation as ***the sum of inversed*** distances to all other nodes as an alternative of the ***inversed of the sum*** of distances to all other nodes. The equation would then be:

$$Closeness\ (i) = \sum_j \frac{1}{d_{ij}}$$

Modular overlap. It is the participation of node i in effective number of modules a

$$O[i] \coloneqq n_a(\{d_i[a]\},$$

Bridgeness measure : For links it is referred as the bridge whose deletion would increase the number of components of the graph. This measure for node *i* between two (a and b) or more modules relative to the other overlapping nodes can be computed as follows:

$$B[i] = \sum_{a=1}^{m} \sum_{b \neq a, b=1}^{m} B[a][b][i]$$

Community centrality:

$$O_{ij}(n) = 2\frac{H_i(n)H_i)(n)}{c(n)}$$

where module membership values $H_i(n)$ and $H_j(n)$ were normalized to the centrality and $O_{ij}(n)$ is proportional to $H_i(n)$ and $H_j(n)$, and c(n) is the centrality of node *n,* while the factor of *2* implies that bidirections are taken into account between the modules.

2: Node classification and annotation in phenotypic multitype OS Networks.

Classification of Nodes: Nodes have been classified into different types according to their pattern of intra- and inter-modular connectivity, thus offering a cartographic representation of networks and measuring through well-known participation coefficients (how much a node overlaps with different modules) and z-scores (how well connected a node is with other nodes in the module it belongs to) (Guimera' et al, 2007; Mihalik et al, 2011).

Non-hub nodes:

1. **Ultra-peripheral:** with all their links within their module
2. **Peripheral:** with most links within their module
3. **Connector:** with many links to other modules
4. **Kinless:** with links homogeneously distributed among all modules

Hub nodes:

5. **Provincial:** with bulk of connections within their module
6. **Connector:** with many connections to most of the other modules
7. **Kinless:** with links homogeneously distributed among all modules.

The nodes classified in gene-gene co-expression networks show various evidences. In Tp there appear non-hub kinless genes with multiple genes, see for instance TGM2, NPPB, and IL1A along with other genes involved significantly in biological processes related to homeostasis. Also, non-hub connector genes showed significant involvement in diverse biological processes such as blood vessel development, ECM, and negative regulation of proliferation. No annotations were retrieved for very few peripheral and ultra-peripheral nodes. Cp and Ip appear rich in peripheral nodes. Ip contains 50% of genes classified as non-hub kinless and non-hub connector genes, showing very little involvement in biological processes. Non-hub kinless genes COL1A2, ARHGDIB showed functions significantly related to the Rho Protein signal transduction, while non-hub connector genes CCND1, LAMA5, TSPAN31, GAL showed function related to regulation of cell proliferation. The peripheral nodes in Ip show important functionality at a genetic level in relation with ECM and regulation of cell adhesion, and also included are co-expressed NDN and GAS1 genes involved in many different biological processes such as developmental growth. Similarly, in Cp colony the co-expressed genes show in particular involvement in cell adhesion and

response to extracellular stimulus. In Pp, non-hub kinless genes amount to 36.5%, and genes PLAT, ENPP2, LAMA5, GAS1 annotate for cell motion, while very few genes from the 31.5% of non-hub connectors show involvement in biological processes. Genes IER3, IL1B, RRAGA, IL1A are predominantly related to both apoptotic and non-apoptotic functions. Peripheral nodes show co-expressed genes mostly showing variety of biological processes, such as estrogen stimulus, steroid hormone stimulus, and positive regulation of cell cycle. The peripheral genes CCND1, TXNIP, CCND2 involved in the above processes show high differential expression in OS cell lines.

3. Variation in PPINs.

Mutations. Proteins containing missense mutations showed no interactions except for FARP1 in Cp, whereas the COL protein family showed interactions and substitution of unknown type (SM Fig. 6). The seed proteins in each phenotype listed in Supplementary Table 4 contain different types of variations: missense, deletion – frame shift, unknown and substitution – coding silent showed no significant GO molecular function terms with adj p-value = 0.01 for Tp, and contains variations in Ip, Cp, and Pp in proteins having molecular functions related to platelet-derived growth factor binding, ECM structural constituent and growth factor binding. Both PHLDA1 and PPP2R2B, which have missense mutation along with IL1A with coding silent variation, are involved in biological processes related to cell death in Tp. Both FARP1 and FBN1 proteins, which show mutations, were DE in Cp. DEGs in Pp contained missense mutation in EEF1A2, STMN2, deletion- frameshift in ENPP2 and substitution – coding silent in IL1A were seen involved in anti-apoptotic activity (no enrichment was found for proteins having mutations in Cp and Ip.

DE Transcription factors in Cp. SP7: This gene encodes a member of the Sp subfamily of Sp/XKLF transcription factors. Sp family proteins are sequence-specific DNA-binding proteins characterized by an amino-terminal trans-activation domain and three carboxy-terminal zinc finger motifs. This protein is a bone specific transcription factor and is required for osteoblast differentiation and bone formation**. MYC:** The protein encoded by this gene is a multifunctional, nuclear phosphoprotein that plays a role in cell cycle progression, apoptosis and cellular transformation. It functions as a transcription factor that regulates transcription of specific target genes. **HOXB9:** This gene is a member of the Abd-B homeobox family and encodes a protein with a homeobox DNA-binding domain. It is included in a cluster of homeobox B genes located on chromosome 17. The encoded nuclear protein functions as a sequence-

specific transcription factor that is involved in cell proliferation and differentiation. Increased expression of this gene is associated with some cases of leukemia, prostate cancer and lung cancer. **FOX:** (Forkhead box) proteins are a family of transcription factors that play important roles in regulating the expression of genes involved in cell growth, proliferation, differentiation, and longevity.

4. 1st PPIN: phenotypic characteristics.

The 1st order PPIN networks comprised many overlapping modules in each OS phenotype with hubs as core nodes of the modules (see Supplementary file 6, molecular function annotations in modules). The Pp 1st order PPIN network is composed of 1447 proteins with on average 70.04 neighbors and higher CC, as compared with the other phenotypes. The central proteins of overlapping modules are particularly interesting, because of the crosslinks they enable (exerting global effects) and since they characterize specific module functions (Segal et al, 2003;.Hydbring and Larsson, 2010). By looking at 26 overlapping modules found in Tp, CDK2, KISS1, TGM2 and IL1A showed processes predominantly related to nucleotide binding, enzyme regulator activity, cell cycle arrest and immune system process (Suppl Mat Table 3A). We note the presence of proteins corresponding to cell cycle genes, such as CDK2 and MYC, playing role in suppressing cellular senescence(Lawrenson et al, 2015), along with TP53, widely known and involved in cell cycle arrest, apoptosis and DNA repair.

Notably, the highly down-regulated NPPB along with the thrombospondin family (THBS2 and THBS3) mediating cell-to-cell and cell-to-matrix interactions and inhibiting tumor growth and angiogenesis, present modular activity of OS relevance from cancer-associated fibroblasts, already observed in lung and epithelial ovarian cancer (Aujollet et al, 2010; Bertin et al, 1997; Zhang et al, 2013). In Ip, CUL3 and CDK6 proteins appearing in multiple modules are involved in enzyme regulator activity along with protein and DNA binding. Then, PLAT, the KRT family proteins (KRT8, KRT31), and CXCL1 involve modularity related to Pyrimidine deoxy-ribonucleotide binding and signal transducer activities. Other modules refer to cytoskeletal protein binding, protein tyrosine kinases activity, and ECM structural constituent (Suppl Mat Table 3B). In general, invasiveness depends on aberrant activities transcription factors, protein kinases and phosphatases. We have noticed in modules both KISS1 and KISS1R, possibly indicating mediation effects, in particular promoting tumor growth (Savvidis et al, 2010;Silva, 2004). Moreover, the modular presence of the hyperactive Jak-Stat protein family in both Ip and Cp suggests relevance of enhancing motility, migration and invasiveness processes, already known in different cancers. Note that proliferation signaling often starts with growth factors activating receptor tyrosine

kinases, and subsequently other cascading pathways depending on the GPCR activation (Chandhanayingyong et al, 2012).

In Cp, modularity mainly depended on MYC and NFKB, and in other cases on promoter binding. Both GRB2 and MYC suggest OS involvement of the MAPK-ERK pathway (note that targeted inhibition of EFGR reduces colony forming, invasion and motility in OS cell lines (Tari et al, 1999; Teixeira and Reed, 2013). Modularity around JUN, MAFB and STAT4 proteins imply transcriptional regulation activity, whereas other proteins in the Stat family (STAT1, STAT3), FGFR3 and KRT8 underlie the modular involvement of receptor signaling protein activity (Supplementary Table 3C). Notably, Pp intra-modular proteins like for instance UBC, CDK2, CDK6, CUL1 and CUL3, and the F box family (FBXO4, FBXO5) along with CTRC and SNURF proteins, are mainly related to ubiquitin-protein ligase activity. The ubiquitin ligases are key players in cell cycle control and presence of proteins in Pp is relevant with respect to aggressive OS (Mc Gary et al, 2002; Randle and Laman, 2016). PDGFRA, PDGFC and PLAT formed modular activity and showed involvement in function related to platelet-derived growth factor receptor binding, ECM structural constituent and endopeptidase activity (see Suppl data S5). Core proteins PDGFRA, Fbox family proteins, CDK2, CUL1, along with proteins present in other modules such as NFKB1, KISS1R, PPP2CA EGFR, are known to enhance proliferation in cancer (Mongre et al, 2014; Forman et al, 2005) (Suppl Mat Fig. 4C).

Supplementary References

Aujollet N, Meyer M, Cailliod R, Combier F, Coignet Y, Campard S, et al.(2010) High N-terminal pro-B-type natriuretic peptide: a biomarker of lung cancer? *Clin Lung Cancer*. 11(5): 341–5.

Bertin N, Clezardin P, Kubiak R, Frappart L. (1997) Thrombospondin-1 and -2 messenger RNA expression in normal, benign, and neoplastic human breast tissues: correlation with prognostic factors, tumor angiogenesis, and fibroblastic desmoplasia. *Cancer Res* 57(3): 396–9.

Chandhanayingyong C, Kim Y, Staples JR, Hahn C, Lee FY (2012) MAPK/ERK Signaling in Osteosarcomas, Ewing Sarcomas and Chondrosarcomas: Therapeutic Implications and Future Directions. *Sarcoma* 2012: 404810 .

Dijkstra EW (1959) A note on two problems in connexion with graphs. *Numer Math* 1(1): 269– 71.

Forman JJ, Clemons PA, Schreiber SL, Haggarty SJ, Eisenberg E, Levanon E et al (2005)

SpectralNET – an application for spectral graph analysis and visualization. *BMC Bioinform* 6: 260.

Freeman LC (1977). A set of measures of centrality based on betweeness. *Sociometry* 40: 35–40.

Guimerà R, Nunes Amaral LA (2005) Functional cartography of complex metabolic networks. *Nature* 433 (7028): 895–900.

Guimerà R, Sales-Pardo M, Amaral LA (2007) Classes of complex networks defined by role-to-role connectivity profiles. *Nat Phys* 3(1) 63–9.

Hydbring P, Larsson LG (2010) Tipping the balance: Cdk2 enables Myc to suppress senescence. *Cancer Res* 70(17) 6687–91.

Lawrenson K, Grun B, Lee N, Mhawech-Fauceglia P, Kan J, Swenson S, et al. (2015) NPPB is a novel candidate biomarker expressed by cancer-associated fibroblasts in epithelial ovarian cancer. *Int J Cancer* 136(6): 1390–401.

McGary EC, Weber K, Mills L, Doucet M, Lewis V, Lev DC, et al (2002) Inhibition of platelet-derived growth factor-mediated proliferation of osteosarcoma cells by the novel tyrosine kinase inhibitor STI571. *Clin Cancer Res* 8(11): 3584–91.

Mihalik A, Csermely P (2011) Heat shock partially dissociates the overlapping modules of the yeast protein-protein interaction network: a systems level model of adaptation. *PLoS Comp Biol* 7(10): 24.

Mongre RK, Sodhi SS, Ghosh M, Kim JH, Kim N, Sharma N et al (2014) A New Paradigm to Mitigate Osteosarcoma by Regulation of MicroRNAs and Suppression of the NF-κB Signaling Cascade. *Dev Reprod* 18(4): 197–212.

Opsahl T, Agneessens F, Skvoretz J (2010) Node centrality in weighted networks: Generalizing degree and shortest paths. *Soc Networks* 32(3): 245–51.

Randle SJ, Laman H (2016) F-box protein interactions with the hallmark pathways in cancer. *Semin Cancer Biol* 36**:** 3–17.

Savvidis C, Papaoiconomou E, Petraki C, Msaouel P, Koutsilieri M (2010) The role of KISS1/KISS1R system in tumor growth and invasion of differentiated thyroid cancer. *Anticancer Res* 35(2): 819–26.

Segal E, Shapira M, Regev R, Pe'er D, Botstein D, Koller D, et al. (2003) Module networks: identifying

regulatory modules and their condition-specific regulators from gene expression data. *Nat Genet* 34(2): 166–76.

Silva D (2004) Signaling pathways responsible for cancer cell invasion as targets for cancer therapy. *Curr Cancer Drug Targets* 4(4): 327–36.

Tari AM, Hung MC, Li K, Lopez-Berestein G (1999). Growth inhibition of breast cancer cells by Grb2 downregulation is correlated with inactivation of mitogen-activated protein kinase in EGFR, but not in ErbB2, cells. *Oncogene* 18(6): 1325–32

Teixeira LK, Reed SI (2013) Ubiquitin ligases and cell cycle control. Annu Rev Biochem 82: 387–414.

Watts DJ, Strogatz SH (1998) Collective dynamics of small-world networks. *Nature* 393(6684) 440–2.

Weisstein EW. Adjacency Matrix. Definition from MathWorld--A Wolfram Web Resource.

Zhang Y, Tang YJ, Li ZH, Pan F, Huang K, Xu GH (2013) KiSS1 inhibits growth and invasion of osteosarcoma cells through inhibition of the MAPK pathway. *Eur J Histochem* 57(4) e30

SM Tables

SM Table 1. Cell Line specific evidences (DEGs) (top), followed by topological properties of DE miRNA-gene target networks**. A)** Proteins coding (seed proteins) for DEGs showing interactions in Human proteome (see methods) and n. of proteins and interactions in their 1st order networks composed of overlapping modules. **B)** Proteins for DEGs showing interactions in Human proteome (see methods) and n. of proteins and interactions in their 1st order networks composed of overlapping modules.

**Tumorigenicity**	**Non-Tumorigenicity**	**Colony forming ability**	**Non -colony forming abilility**	**Invasive/ migratory potential**	**Non-invasive**	**Proliferation capacity**	**Non Proliferating**
HOS, MHM, OHS and OSA	IOR/MOS, IOR/OS10 and IOR/OS18	MG-63, HOS and OSA	HAL, IOR/MOS, IOR/OS9, IOR/OS14 and ZK58	HAL, HOS, OSA, U2OS	IOR/MOS, IOR/SARG, MG63 and ZK-58	HOS, MHM, OHS and OSA	HAL, IOR/MOS, IOR/OS9, IOR/OS14 and KPD

**A. gene – miRNA target networks**	Nodes	Links	Clustering Coefficient	Characteristic Pathlength	Average number of neighbors	Network heterogeneity	Diameter	Density
**Tumorigenic**	281	2381	0.28	2.759	14.512	1.200	5	0.052
**Colony forming**	309	2372	0.261	2.876	13.638	1.231	5	0.044
**Invasive**	212	1168	0.243	2.913	9.377	1.124	8	0.044
**Proliferative**	168	658	0.223	3.059	6.476	1.303	8	0.039

**B. 1st order PPIN**	Nodes	Links	N. of modules	Clustering Coefficient	Characteristic Path length	Network heterogeneity	Diameter	Density	Seed nodes	Links
**Tumorigenic**	2032	58254	27	0.37	2.25	1.171	6	0.028	102	20
**Invasive**	1737	54097	24	0.38	2.24	1.122	6	0.036	82	32
**Colony forming**	2324	65197	17	0.36	2.25	1.259	6	0.024	104	29
**Proliferation**	1447	50675	26	0.43	2.17	1.004	5	0.048	56	9

SM Table 2. Biological processes of seed proteins showing interactions in PPI networks A) Tp; B) Cp; C) Ip; D) Pp.

**A) Tp**			
**GO biological process complete**	**N. of proteins involved**	**Fold Enrichment**	**P value**
Collagen catabolic process	4	59.58	4.89E-03
Collagen metabolic process	4	51.53	8.69E-03
Multicellular organismal macromolecule metabolic process	4	48.27	1.12E-02
Multicellular organism metabolic process	4	41.00	2.14E-02
Multicellular organism catabolic process	4	53.71	7.38E-03

B) Cp			
**GO biological process complete**	**N. of proteins involved**	**Fold Enrichment**	**P value**
Focal adhesion assembly	3	90.40	4.32e-02
Cell-matrix adhesion	5	30.13	5.24e-03
Cell-substrate adhesion	5	22.05	2.39e-02
Cell-substrate adherens junction assembly	3	90.40	4.32e-02
Collagen catabolic process	4	45.20	1.56e-02
Collagen metabolic process	4	39.09	2.77e-02
Multicellular organismal macromolecule metabolic process	4	36.62	3.58e-02
Single-multicellular organism process	25	3.43	6.32e-08
Multicellular organismal process	26	2.98	3.00e-07
Multicellular organism catabolic process	4	40.74	2.35e-02
Regulation of rho protein signal transduction	5	32.58	3.58e-03
Regulation of ras protein signal transduction	5	19.13	4.75e-02
Regulation of cellular process	26	1.83	3.98e-02
Regulation of biological process	27	1.81	1.27e-02
Regulation of response to stimulus	16	3.20	3.35e-02
Extracellular matrix organization	9	21.20	1.91e-06
Extracellular structure organization	9	21.13	1.97e-06
Response to acid chemical	6	14.91	2.08e-02
Response to stimulus	23	2.21	1.80e-02
Morphogenesis of an epithelium	7	12.50	8.67e-03
Tissue morphogenesis	9	12.86	1.44e-04
Tissue development	11	5.32	1.99e-02
Anatomical structure development	22	3.23	3.02e-05
Developmental process	22	3.15	5.03e-05
Anatomical structure morphogenesis	16	5.64	1.09e-05
Single-organism developmental process	22	3.23	3.00e-05
Tube development	8	10.29	5.56e-03
Multicellular organism development	22	3.54	4.96e-06
Regulation of body fluid levels	7	10.12	3.47e-02
Cell migration	9	8.51	4.75e-03
Cell motility	9	7.73	1.05e-02
Movement of cell or subcellular component	11	6.18	4.59e-03

Localization of cell	9	7.73	1.05e-02
Organ morphogenesis	9	7.58	1.24e-02
Animal organ development	18	4.66	1.12e-05
System development	21	3.89	3.42e-06

C) Ip

Go biological process complete	**N. of proteins involved**	**Fold enrichment**	**P value**
Extracellular matrix organization	7	15.94	1.76e-03
Extracellular structure organization	7	15.89	1.80e-03
Single-organism process	29	1.63	2.80e-02
Wound healing	7	10.52	2.76e-02
Response to wounding	8	9.99	7.20e-03
Tissue development	12	5.61	3.52e-03
Anatomical structure development	19	2.70	3.04e-02
Developmental process	19	2.63	4.57e-02
System development	17	3.04	3.10e-02
Multicellular organism development	18	2.80	4.28e-02
Single-multicellular organism process	22	2.92	3.31e-04
Multicellular organismal process	22	2.43	1.08e-02
Biological regulation	28	1.72	2.96e-02

D) Pp

**GO biological process complete**	**N. of proteins involved**	**Fold Enrichment**	**P value**
**Extracellular matrix organization**	6	25.62	5.60E-04
**Extracellular structure organization**	6	25.53	5.71E-04

SM Table 3: Mutation in OS patients in PPI–miRNA networks.

Gene	Type	Amino acid mutation	Somatic status	CDS Mutation
**Tp**				
**COL6A3**	Substitution - coding silent	p.L1194L	Confirmed	c.3582G>A
**COL6A3**	Substitution - coding silent	p.L988L	Confirmed	c.2964G>A
**IL1A**	Substitution - coding silent	p.V122V	Confirmed	c.366G>A
**PHLDA1**	Substitution - Missense	p.T277M	Confirmed	c.830C>T
**PPP2R2B**	Substitution - Missense	p.S78N	Confirmed	c.233G>A
**Cp**				
**OLFM1**	Substitution - coding silent	p.G385G	Confirmed	c.1155G>A
**STEAP3**	Substitution - coding silent	p.S439S	Confirmed	c.1317A>G
**LEPREL1**	Unknown	p.?	Confirmed	c.1549-8_1549-7insC
**LEPREL1**	Unknown	p.?	Confirmed	c.1549-8_1549-7insT
**FBN1**	Substitution - coding silent	p.G2811G	Confirmed	c.8433G>A
**FBN1**	Substitution - Missense	p.V2823E	Confirmed	c.8468T>A
**COL1A2**	Unknown	p.?	Confirmed	c.2133+3_2133+4insGT
**FARP1**	Substitution - Missense	p.A854V	Confirmed	c.2561C>T
**FARP1**	Substitution - Missense	p.A885V	Confirmed	c.2654C>T
**COL6A3**	Substitution - coding silent	p.L1194L	Confirmed	c.3582G>A
**COL6A3**	Substitution - coding silent	p.L988L	Confirmed	c.2964G>A
**COL4A1**	Unknown	p.?	Confirmed	c.652-5C>A
**EEF1A2**	Substitution - Missense	p.T104M	Confirmed	c.311C>T
**FAM46A**	Substitution - coding silent	p.L240L	Confirmed	c.720T>C
**FAM46A**	Substitution - coding silent	p.L321L	Confirmed	c.963T>C

Ip				
**COL1A2**	Unknown	P.?	Confirmed	c.2133+3_2133+4insGT
**EEF1A2**	Substitution - Missense	p.T104M	Confirmed	c.311C>T
**COL4A1**	Unknown	p.?	Confirmed	c.652-5C>A
**Pp**				
**ENPP2**	Deletion - Frameshift	p.V697fs*2	Confirmed	c.2088_2097del10
**ENPP2**	Deletion - Frameshift	p.V670fs*2	Confirmed	c.2007_2016del10
**COL1A2**	Unknown	p.?	Confirmed	c.2133+3_2133+4insGT
**STMN2**	Substitution - Missense	p.E115K	Confirmed	c.343G>A
**IL1A**	Substitution - coding silent	p.V122V	Confirmed	c.366G>A
**EEF1A2**	Substitution - Missense	p.T104M	Confirmed	c.311C>T
**COL4A1**	Unknown	p.?	Confirmed	c.652-5C>A

SM Table 4. Shared pathways (32) in all OS phenotypes and corresponding DEGs.

Pathway Terms	Tp	Ip	Cp	Pp
**a6b1 and a6b4 Integrin signaling**	CD9, IL1A, LAMA4	LAMA5	LAMA5	IL1A, LAMA5, LAMC2
**amb2 Integrin signaling**	PLAU, RHOA, THY1	PLAT, RHOA	CTGF, PLAT	PLAT
**AP-1 transcription factor network**	PLAU	CCND1, COL1A2	CCND1, COL1A2, GJA1, MYC	CCND1, COL1A2
**ATF-2 transcription factor network**	CDK4, PLAU	CCND1, PDGFRA	CCND1, CSRP2	CCND1

**Beta1 integrin cell surface interactions**	COL6A1, COL6A2, COL6A3, ITGA11, LAMA4, PLAU, TGFBI, TGM2, THBS2	COL11A1, COL1A2, COL4A1, COL4A2, LAMA5, MDK, TGFBI	COL1A2, COL3A1, COL4A1, COL6A3, ITGA11, LAMA5, TGM2, THBS1	COL1A2, COL4A1, ITGA11, LAMA5, LAMC2
**Beta2 integrin cell surface interactions**	PLAU, TGFBI, THY1	PLAT, TGFBI	PLAT	PLAT
**Beta3 integrin cell surface interactions**	LAMA4, PLAU, SDC4, TGFBI, THY1	COL1A2, COL4A1, COL4A2, TGFBI	COL1A2, COL4A1, IBSP, THBS1	COL1A2, COL4A1
**Caspase Cascade in Apoptosis**	KRT18	ARHGDIB, KRT18	ARHGDIB	ARHGDIB
**C-MYB transcription factor network**	ADORA2B	CCND1, CDK6, COL1A2	CCND1, COL1A2, MYC	CCND1, COL1A2
**Coregulation of Androgen receptor activity**	FHL2	CCND1, CDK6, SNURF	CCND1	CCND1, SNURF
**CXCR4-mediated signaling events**	ITGA11, RHOA, RHOB, RHOC	RHOA, RHOB, RHOC	ITGA11	ITGA11
**Direct p53 effectors**	DKK1, IGFBP3, NDRG1	CX3CL1	STEAP3, TAP1	NDRG1
**E-cadherin signaling in the nascent adherens junction**	RHOA	CCND1, RHOA	CCND1, CTTN	CCND1
**Endothelins**	RHOA	COL1A2, RHOA	COL1A2, COL3A1	COL1A2
**FOXM1 transcription factor network**	CDK4, GAS1, LAMA4	CCND1, GAS1	CCND1, MYC	CCND1, GAS1
**Glucocorticoid receptor regulatory network**	KRT17, SGK1	KRT17	KRT17	KRT17

**IL4-mediated signaling events**	THY1	COL1A2	COL1A2	COL1A2
**Integrin-linked kinase signaling**	RHOG	CCND1, MYL9, RHOG	CCND1	CCND1
**Integrins in angiogenesis**	COL6A1, COL6A2, COL6A3, RHOA	COL11A1, COL1A2, COL4A1, COL4A2, COL8A1, RHOA	COL10A1, COL16A1, COL1A2, COL3A1, COL4A1, COL6A3	COL1A2, COL4A1
**Neurotrophic factor-mediated Trk receptor signaling**	RHOA, RHOG	CCND1, RHOA, RHOG	CCND1	CCND1
**Noncanonical Wnt signaling pathway**	RHOA	RHOA	CTHRC1	CTHRC1
**Presenilin action in Notch and Wnt signaling**	DKK1	CCND1	CCND1, MYC	CCND1
**Regulation of nuclear beta catenin signaling and target gene transcription**	DKK1	CCND1, CCND2	CCND1, MYC	CCND1, CCND2
**Regulation of nuclear SMAD2/3 signaling**	CDK4	COL1A2	COL1A2, MYC	COL1A2
**Regulation of retinoblastoma protein**	CDK4	CCND1, CCND2, CDK6	CCND1	CCND1, CCND2
**Regulation of RhoA activity**	RHOA	ARHGDIB, RHOA	ARAP3, ARHGDIB, DLC1, FARP1	ARHGDIB
**Signaling events mediated by focal adhesion kinase**	RHOA	CCND1, RHOA	CCND1	CCND1

**Syndecan-1-mediated signaling events**	COL6A1, COL6A2, COL6A3	COL11A1, COL1A2, COL4A1, COL4A2, COL8A1, LAMA5	COL10A1, COL16A1, COL1A2, COL3A1, COL4A1, COL6A3, LAMA5	COL1A2, COL4A1, LAMA5
**Validated targets of C-MYC transcriptional activation**	CDK4	CCND2, SERPINI1	MYC	CCND2
**Validated targets of C-MYC transcriptional repression**	DKK1, NDRG1, TMEFF2	CCND1, COL1A2	CCND1, COL1A2, MYC, SFRP1	CCND1, COL1A2, NDRG1
**Validated transcriptional targets of AP1 family members Fra1 and Fra2**	DCN, MGP, PLAU	CCND1, COL1A2, DCN	CCND1, COL1A2, GJA1	CCND1, COL1A2, DCN
**Wnt signaling network**	DKK1, IGFBP4	SOST	CTHRC1, SOST	CTHRC1

SM Table 5: Overlapping modular organization 1st order PPI networks characterizing each phenotype on the basis of molecular functions assigned to each module (based on molecular function of central proteins of the modules with significant p-value for phenotypes A) Tp; B) Ip; C) Cp; D) Pp (complete list in Supplementary file 3).

A) Tp

Phenotype	module Id	Module name	N. of nodes	Effective n. of nodes	Module size (sum of module assignment values)	Ten overlapping core proteins of module	Molecular function Description	Adj. P value
Tumorigenic 1st order PPI	**Module 1**	UBC	2026	582.2	1.68E+07	UBC;ELAVL1;FN1;APP;TP53;SUMO2;CTNNB1;HSP90AA1;CDK2;ESR1;	Nucleotide binding	4.98E-02
	**Module 2**	APP	2025	532.8	1039330	APP;UBC;CUL3;KISS1;HSP90AA1;A2M;FBLN1;HSP	Enzyme regulator activity	2.70E-02

					A5;CTNNB1; YWHAZ;		
**Module 3**	ADM	2014	18.43	61658.57	ADM;MME;CFH;GPR182; RAMP2;EDN1;EEF1A1;NPPC;HSP90AA1;TUBA1A;	Hormone activity	1.09E-03
**Module 4**	ARRB2	1990	14.69	52401.83	ARRB2;CALCRL;RAMP2;TGFBR3;CXCL12;SDC3;CDK4;ARHGAP17;CDC42;HDAC2;	Alcitonin gene-related polypeptide receptor activity	2.95E-02
**Module 5**	ITGA4	1999	36.38	69721.17	ITGA4;THBS2;HDAC4;THBS3;ITGB1;ADAM2;CD53; FCGR2C;MMP2;NPPB;	Protein binding	3.28E-02
**Module 6**	SUMO1	1996	35.22	70296.2	SUMO1;PDGFC;FZD8;MZF1;OBSCN;SYDE2;MAF; IKBKB;SDF2; EGR1;	Platelet-derived growth factor receptor binding	1.47E-03
**Module 7**	JUN	1987	19.83	31316.88	JUN;PLXNA2; STAT4;MAF; PDZD2;CYP19A1;EGR1;SRF;BBS7;VAV1;	Organ development	4.31E-02
**Module 8**	CAND1	1988	26.98	34297.27	CAND1;COL19A1;COL3A1; CD36;COL4A2;MMP2;CALML3;TF;F7;ECT2;	Extracellular matrix structural constituent	9.59E-04
**Module 9**	GRB2	1990	28.04	42610.52	GRB2;SGCA; FGF7;DAG1; CCL5;KRT34; AMBP;ADAM12;CFH;SS18;	Protein binding	4.04E-02

	**Module 10**	PPP2CA	1985	17.22	26152.81	PPP2CA;KISS1R;PIR;SCHIP1;IER5;PTN;PXDN;MYO9A;RHOB;PAXIP1;	No annotations	
	**Module 11**	MYC	1983	34.86	57936.12	MYC;ROBO2;PLAU;ITGA1;SLIT2;MYO9A;NUCB1;UBQLN4;PDZD2;MRPL53;	Signaling process	1.89E-04
	**Module 12**	TUBA1A	1983	27.05	30697.15	TUBA1A;NPPC;MDK;SDC3;STARD13;KRT31;NPR3;NCK1;GLA;AFG3L2;	Regulation of multicellular organism growth	3.87E-02
	**Module 13**	CUL2	1966	21.03	24804.75	CUL2;LOXL1;ATXN1;MLPH;ELN;APOD;ARHGEF4;PEPD;OSGEP;CTSD;	No annotations	
	**Module 14**	CUL1	1995	34.72	40013.82	CUL1;SLC7A9;TGM2;MYBPC3;RPRM;MMP2;FBXO2;SORL1;CDKN1C;GPATCH4;	Cell cycle arrest	6.19E-03
	**Module 15**	IL1A	1926	18.27	32310.1	IL1A;TRAF6;IL1R2;CAPN1;IRAK1;HAX1;TOLLIP;TNFRSF13B;NFKBIE;MYD88;	Immune system process	8.89E-05
	**Module 16**	SFN	1958	14	18714.41	SFN;ANPEP;TM4SF1;PKP3;ZFYVE19;ARHGEF16;DTX2;ARHGEF17;TRIM25;RHPN2;	Rho guanyl-nucleotide exchange factor activity	2.15E-02

	**Module 17**	HNRNPC	1961	19.52	20789.9	HNRNPC;LMO3;PDGFB;CCDC85B;MDFI;MBIP;IFIT1;KHDRBS3;ECT2;POLR2K;	No annotations	
	**Module 18**	CANX	1967	13.13	14957.46	CANX;AMBN;BGN;TF;SERPINF2;SUMO2;GRN;CD63;CD9;TOR1A;	Zinc ion binding	4.92E-02
	**Module 19**	NUDC	1955	9.52	13789.66	NUDC;LXN;CPA4;GDI2;EEF1A1;KIAA0101;ARIH1;RANBP2;RANGAP1;PRKACA;	Zinc ion binding	4.92E-02
	**Module 20**	DCUN1D1	1958	7.87	13797.48	DCUN1D1;KIAA1199;GDI1;EEF1A1;SOCS5;SCRIB;CALM1;DAZAP2;SMAD2;GLRX3;	Protein binding	3.56E-02
	**Module 21**	CAV1	1970	14.8	18177.69	CAV1;RAB27B;UNC13D;MLPH;WNT3A;IGFBP3;KCNA5;ACVRL1;GJB2;PRNP;	Microtubule binding	3.39E-02
	**Module 22**	PAXIP1	1942	15.8	26996.5	PAXIP1;SCAMP5;FBLN5;PLAU;ATN1;GFI1B;A2M;DNAJA1;EP300;TPM3;	Enzyme binding	2.86E-02
	**Module 23**	DCN	1951	10.57	19327.41	DCN;SFTPD;EGFR;FLNA;BRCA1;WISP1;ELN;DPT;MMP3;GJA1;	Cytoskeletal protein binding	3.28E-02
	**Module 24**	NFIX	1863	6.51	15516.87	NFIX;ALKBH3;RPS27A;ISG15;HDAC1;	Transcription regulator activity	1.44E-02

					RB1;POLR2E;SUPT16H;RPS6KA1;SKI;		
	**Module 25**	LIG4	1892	10.49	13726.47	LIG4;CTSK;SPARC;VKORC1;SERPINB13;EEF1A1;CREBBP;UNC119;PRKDC;XRCC6;	Protein C-terminus binding	3.31E-02
	**Module 26**	GRN	1888	11.65	14407.02	GRN;DLK1;HK3;SLPI;HOXA1;ZNF8;ELANE;FRAT1;GFI1B;GAS1;	No annotations	
	**Module 27**	BMP2	1845	5.52	7984.249	BMP2;MGP;SMURF1;ACTR2;ACVR1;BMP1;SMURF2;SMAD4;SMAD1;COL2A1;	SMAD binding	1.30E-09

B) Ip

**Phenotype**	**module Id**	**Module name**	**N. of nodes**	**Effective n. of nodes**	**Module size (sum of module assignment values)**	**Ten overlapping core proteins of module**	**Molecular function Description**	**Adj P value**
**Invasive 1st order PPI network**	**Module 1**	UBC	1730	528.4	1.14E+07	UBC;FN1;APP;GRB2;CDK2;ESR1;CUL3;CDK6;TP53;EEF1A1;	Protein binding	2.42E-02
	**Module 2**	APP	1730	404.82	635483.5	APP;UBC;KISS1;CUL3;BGN;YWHAZ;COPS5;HSP90AA1;SERPINA3;HSPA5;	Enzyme regulator activity	9.46E-03
	**Module 3**	FN1	1728	461.28	503463.2	FN1;SERPINA3;EGFR;DCN;SUMO2;APP;ERBB2;RAC1;UBC;N	No annotations	

						FKB1;		
	**Module 4**	ELAVL1	1723	223.17	418983.9	ELAVL1;GAL;SUMO2;TP53;CDK6;CUL3;CUL1;CDC42;GRB2;KIAA0101;	DNA binding	1.81E-02
	**Module 5**	HSP90AA1	1694	41.34	87355.91	HSP90AA1;G6PC;TRIM74;FBXL2;METTL22;EPHB6;STARD13;ACVR2B;CNKSR1;MMP2;	Enzyme binding	3.48E-04
	**Module 6**	YWHAZ	1696	50.53	60933.57	YWHAZ;LUM;ADAM22;PKP3;NOXA1;GP5;GP9;CBY1;GP1BB;ARHGEF18;	No annotations	
	**Module 7**	MDK	1654	31.48	68042.58	MDK;TUBA1A;GPC2;ACTG1;NCL;RPL18A;STAT1;JAK1;MAPK6;JAK2;	No annotations	
	**Module 8**	NEDD8	1684	21.04	24518.74	NEDD8;CTRC;RHOBTB2;FBXO4;ARHGEF4;DCTPP1;KCTD13;UBE2F;GDI1;HRAS;	Nucleotide binding	1.20E-02
	**Module 9**	KRT8	1684	22.17	32294	KRT8;QRSL1;TCHP;PLAT;STAM2;MOG;CLN5;FGFR3;DEDD;KRT31;	Pyrimidine deoxyribonucleotide binding	2.65E-02
	**Module 10**	PXN	1692	20.82	42156.5	PXN;COL8A1;COL8A2;VCL;COL5A2;UBQLN4;KLHL12;ITGA2;ITGA1;PTPRZ1;	No annotations	
	**Module 11**	CAND1	1687	26.2	30587.47	CAND1;COL19A1;COL3A1;COL6A2;COL4A2;CD36;CALML3;MMP2;PMEPA1;CEP97;	Extracellular matrix structural constituent	7.42E-04

	**Module 12**	PPP2CA	1687	13.77	17090.78	PPP2CA;KISS1R;PPP2R5B;NPTN;MYO9A;NUBP2;RHOB;NXN;ECT2;INTS1;	No annotations	
	**Module 13**	SUMO1	1692	34.09	33279.24	SUMO1;PDGFC;RBM15B;MZF1;ZNF174;OBSCN;SYDE2;PTPRB;PLAT;ST14;	No annotations	
	**Module 14**	YWHAE	1693	26.19	26706.01	YWHAE;ADH1B;PML;RBMY1A1;ADH4;CBY1;PDE4DIP;ADH1A;FBXO4;GP1BA;	Alcohol dehydrogenase activity. zinc-dependent	1.58E-08
	**Module 15**	A2M	1639	20.2	32214.73	A2M;CELA1;KLKB1;KLK2;PDGFA;PDGFB;TGFBI;SHBG;MMP2;KLK3;	Endopeptidase activity	4.52E-05
	**Module 16**	TRAF6	1682	23.79	35726.51	TRAF6;TNFRSF19;IL1A;TANK;TOLLIP;NDN;NGF;UEVLD;UBE2D4;REXO2;	Receptor binding	1.83E-02
	**Module 17**	ACTB	1689	33.44	24327.51	ACTB;TNNI2;CALM1;SHBG;NCF2;TRIM63;TNNT1;TAF11;TNNC1;CTSG;	Cytoskeletal protein binding	9.70E-06
	**Module 18**	SMAD3	1684	13.4	18023.33	SMAD3;JPH3;CHRD;ZNF8;PLG;ZEB1;ISL1;GIT2;GDNF;UBQLN4;	Sequence-specific DNA binding	4.84E-02
	**Module 19**	CXCL9	1604	13.51	20933.21	CXCL9;CX3CR1;MAPK1;TXN;GNAI2;CX3CL1;STAT1;TNF;GNG2;PLA2G1B;	Signal transducer activity	3.92E-02
	**Module 20**	ANXA2	1644	10.6	9939.128	ANXA2;ENAM;AHSG;COL2A1;COL5A1;PLG;PLAT;MYOC;CCNH;HNF1A;	Structural molecule activity	1.06E-02

	**Module 21**	TNK2	1634	8.63	10230.12	TNK2;ARSE;NDN;SYNJ1;LTK;RASGRF1;BIN1; ALK;RPL18A;MCF2;	Protein tyrosine kinase activity	5.80E-03
	**Module 22**	AHSG	1591	6.99	10270.34	AHSG;AMELX; VKORC1;ENAM;DDB1;AKT1; CDC42;ATM;ATF2;TTN;	Identical protein binding	1.19E-03
	**Module 23**	GRN	1575	8.59	7989.491	GRN;DLK1;ZNF8;GAS1;ELANE; FRAT1;CDK2;KIAA0101;TJP3;PRKDC;	No annotations	
	**Module 24**	SHBG	1575	4.33	5421.888	SHBG;KLK4;SRF;LRP2;EIF3E;ATP5A1;AP1B1;EEF1A1;DSTN;ACTG2;	No annotations	

C) Cp

**Phenotype**	**Module Id**	**Module name**	**N. of nodes**	**Effective n. of nodes**	**Module size (sum of module assignment values)**	**Ten overlapping core proteins of module**	**Molecular function Description**	**Adj P value**
**Colony forming ability 1st**	**Module 1**	UBC	2318	625.89	2.38E+07	UBC;MYC;APP; FN1;ELAVL1;GRB2;CUL3;ESR1;YWHAZ;TP53;	Promoter binding	1.58E-03
	**Module 2**	HSP90AA1	2305	39.97	95549.34	HSP90AA1;G6PC;HOPX;CAMK1G;FGFR3;MMP2;EPHB6;TIE1;IFIT1;ROR2;	Transmembrane receptor protein tyrosine kinase activity	3.89E-06

**Module 3**	HSP90AB1	2288	28.24	37871	HSP90AB1;CYP17A1;PPP2R4;FGFR3;POR;IFIT1;LGALS4;TNNT1;STARD13;MKNK1;	NADPH-hemoprotein reductase activity	3.24E-02
**Module 4**	NEDD8	2272	19.71	29408.03	NEDD8;CTRC;SERPINA3;RHOBTB2;FBXO4;TST;TFE3;ARHGEF4;HIST1H2BG;NDUFS6;	No annotations	
**Module 5**	JUN	2298	24.07	39717.32	JUN;PLXNA2;MAFB;DHX37;HOOK2;FOSB;SHANK1;STAT4;HAP1;MAF;	Transcription regulator activity	4.85E-03
**Module 6**	PPP2CA	2283	15.18	24471.55	PPP2CA;KISS1R;KISS1;PTN;CTTNBP2;NPTN;PPP2R5B;RHOB;DVL3;MYO9A;	Protein phosphatase regulator activity	1.81E-02
**Module 7**	SUMO2	2298	42.15	66807.51	SUMO2;CSRP2BP;CUL4A;WDR5;KALRN;FOXP1;CXXC1;POR;CSRP2;SUPT7L;	Ubiquitin protein ligase binding	3.19E-02
**Module 8**	A2M	2229	23.25	53355.65	A2M;CELA1;IL10;EGLN2;KNG1;KLKB1;TGFBI;KLK2;IL1B;PDGFB;	Growth factor receptor binding	1.16E-03
**Module 9**	MAPK3	2268	11.11	19841.48	MAPK3;CPXM1;PLAT;IGF1;BTBD10;KIAA0101;SOS1;PRDX3;MKNK1;GJA1;	No annotations	
**Module 10**	MFI2	2269	10.32	40449.67	MFI2;PDIA3;DERL3;DERL1;GYPC;DERL2;COL6A3;PDIA2;CDK2;RAC1;	Protein disulfide isomerase activity	3.95E-04

	**Module 11**	DCUN1D1	2243	7.35	15108.29	DCUN1D1;KIAA1199;GDI1;NFKB1;SMAD2;ARHGEF1;EEF1A1;CALM1;MCC;SCRIB;	Promoter binding	2.81E-02
	**Module 12**	FGFR3	2185	12.27	33017.59	FGFR3;CCDC17;CTSK;GPSM3;HSPA8;STAT1;KRT8;PTK2B;HNRNPL;STAT3;	Receptor signaling protein activity	4.00E-02
	**Module 13**	IL1B	2207	8.19	18921.92	IL1B;IL1R2;IL10;MMP2;SQSTM1;SP1;MAP3K3;EGR1;RELA;TRAF6;	Ion binding	1.97E-02
	**Module 14**	SERPINA3	2216	6.36	17816.78	SERPINA3;CTRL;KLK4;NFKB1;ERBB2;PSMA3;POLA1;STK4;CMA1;LMNB1;	Endopeptidase activity	3.61E-03
	**Module 15**	FAM107A	2112	7.678	19782.09	FAM107A;WDR47;VIM;CANX;DCD;TRAF2;PPP2R2A;TADA2A;KRT19;USP15;	Structural constituent of cytoskeleton	4.31E-02
	**Module 16**	SHBG	2135	5.545	8460.812	SHBG;KLK4;SERPINA3;SRF;EEF1A1;LRP2;UBE2O;SNRNP70;ATP5A1;EIF3E;	No annotations	
	**Module 17**	LIG4	2158	10.81	9118.615	LIG4;CTSK;KNG1;SPARC;FGFR3;VKORC1;DOK1;THOC5;EEF1A1;DPM1;	DNA ligase activity	2.91E-02

D) Pp

Phen	Module Id	Module name	Number of	Effective number	Module size	Ten overlapping	Molecular function	Adj P value

		nodes	of nodes	(sum of module assign-ment values)	core proteins of module	Descripti-on	
**Module 1**	UBC	1443	489	0.00059892	UBC; FN1; APP; EEF1A1; CDK2; SUMO2; CUL3; ESR1; GRB2; CUL1;	Ubiquitin protein ligase binding	5.99E-04
**Module 2**	APP	1444	350	386096.8	APP;CUL3;KISS1;UBC;SERPINA3;TRAF6;ACTB;COPS5;MMP24;GRB2;	Enzyme binding	3.29E-02
**Module 3**	FN1	1442	443	409380.5	FN1;SERPINA3;DCN;EGFR;APP;NFKB1;SUMO2;UBC;EEF1A1;BRCA1;	DNA binding	3.07E-02
**Module 4**	YWHAZ	1402	46	113822.5	YWHAZ;EFNB3;RHBDL2;LUM;GP5;GP9;GP1BB;ARHGEF18;ARHGEF16;TCEB1;	Guanyl-nucleotide exchange factor activity	3.80E-02
**Module 5**	HSP90AA1	1425	40	76.021	HSP90AA1; RGS6;G6PC; [illegible]	No Annotations	
	**PLG**		**12**		**PLG;SERPINI1;PLAT;MMP3;MMP7;ACTG1;LAMA3;EP300;ANXA2;TIAM1;**		**6.14E-03**
**Module 6**	ELAVL1	1427	39	99855.51	ELAVL1;ADRA1D;RNF41;SERPINE2;E2F2;IL1B;COL4A5;DOK4;GEMIN2;TRIM8;	No Annotations	
	SHBG		7		SHBG;[illegible]		1.67E-02

	**Module 7**	HSP90AB1	1406	29	29110.91	HSP90AB1;CYP17A1;PPP2R4;TRIM8;STARD13;TAF1D;CHORDC1;RPAIN;ARHGAP1;A2M;	enzyme regulator activity	8.44E-03
	**Module 8**	NEDD8	1408	21	19.719	NEDD8;CTRC;ARHGEF4;RHOBTB2;TST;FBXO4;PIP;GDI1;FBXO5;UBE2K;	Ubiquitin-protein ligase activity	4.44E-02
	**Module 9**	TP53	1409	39	53772.11	TP53;BMP1;DMTF1;S100B;RASGRF1;LAMA4;SMYD2;TRIM8;SNURF;FBXO4;	Receptor binding	1.17E-02
	**Module 10**	SUMO1	1404	28	30.286	SUMO1;PDGFC;MAF;SYDE2;PDGFRA;MAFA;SULT1A1;EGR1;PLAT;STMN2;	Platelet-derived growth factor receptor binding	1.89E-03
	**Module 11**	KIAA0101	1403	34	34415.84	KIAA0101;ELANE;COL4A2;MMP2;GNS;LRP1;THBS1;ITSN1;ANKFY1;SDC1;	Ion binding	2.89E-02
	**Module 12**	UBD	1395	16	18.408	UBD;ENPP2;NPPB;HDAC4;RHOT1;RHOT2;MGRN1;CTTN;EWSR1;SUMO2;	Protein binding	3.72E-02
	**Module 13**	PPP2CA	1401	15	15.403	PPP2CA;KISS1R;PPP2R5B;NPTN;MYO9A;RHOB;ECT2;NXN;I	No annotations	

						ER3;PFDN1;		
	**Module 14**	HSPA5	1417	48	31614.04	HSPA5;HSPA8;DNAJC1;CLTC;PRTN3;PRNP;CTSG;COL7A1;MAP3K8;NFKBIE;	Chaperone binding	1.30E-02
	**Module 15**	NONO	1396	16	12.813	NONO;ZNRD1;HAX1;IRAK3;MAD1L1;SOCS3;DLD;A2M;GDI2;MRPL41;	Interleukin-1 binding	1.39E-03
	**Module 16**	YWHAE	1412	24	20.397	YWHAE;ADH1B;PML;GSTA1;RBMY1A1;IRAK3;FBXO4;GP1BA;RASGRF1;RCN2;	Protein homodimerization activity	4.66E-02
	**Module 17**	PXN	1414	21	15.365	PXN;COL5A2;COL6A3;COL6A2;ITGA1;GFRA1;COL4A2;GDNF;COL5A1;GAB1;	Extracellular matrix structural constituent	5.71E-08
	**Module 18**	A2M	1398	22	33037.53	A2M;CELA1;IL10;TNF;IL1B;TGFBI;KLK2;KLKB1;MMP2;SHBG;	Endopeptidase activity	2.33E-03
	**Module 19**	CKB	1392	27	23053.1	CKB;SERP2;EWSR1;KIAA0947;MYH4;USPL1;VSIG8;SELENBP1;FABP4;KRT34;	Calmodulin binding	4.35E-02

	**Module 20**	PFN1	1388	9	8.142	PFN1;CMA1;NCK1;CDK2;RHOQ;PIK3R1;CUL3;LIG4;ESR1;LRIF1;	Hormone binding	1.86E-02
	**Module 21**	BARD1	1391	10	11.666	BARD1;ZFP64;HAP1;MAFB;CDK2;SELENBP1;PIK3R2;MAPK1;ESR1;RELA;	Transcription factor binding	1.03E-03
	**Module 22**	TUBB	1401	30	14.222	TUBB;GLIS2;CTNNB1;APC;CDH1;CCND1;CPSF1;DISC1;CRYZ;NCAM1;	Enzyme binding	4.45E-03
	**Module 23**	CDKN1A	1387	14	11.743	CDKN1A;TEX11;MAD1L1;STMN2;CCDC85B;COL4A5;CLEC3B;E2F2;CDC42;EGR1;	No annotations	
	**Module 24**	IL1B	1389	10	12.461	IL1B;IL1R2;IL1A;TRAF6;SQSTM1;MMP2;RELA;IL10;SP1;MAP3K3;	Growth factor receptor binding	9.93E-04

SM Table 6. Pathways specific in all OS phenotypes (from corresponding DEGs).

Phenotype	Pathways

**Tumorigenic**	Beta5 beta6 beta7 and beta8 integrin cell surface interactions; Insulin Pathway; ErbB1 downstream signaling;FoxO family signaling; ATR signaling pathway; Urokinase-type plasminogen activator (uPA) and uPAR-mediated signaling; Calcineurin-regulated NFAT-dependent transcription in lymphocytes; Proteoglycan syndecan-mediated signaling events; Glypican 1 network; Signaling events mediated by HDAC Class III; ALK1 signaling events
**Invasive**	PDGF receptor signaling network; Internalization of ErbB1; Glypican 2 network; Retinoic acid receptors-mediated signaling; Circadian rhythm pathway; PDGFR-alpha signaling pathway
**Colony forming**	LKB1 signaling events;IL2 signaling events mediated by PI3K; Ceramide signaling pathway;IL6-mediated signaling events; CD40/CD40L signaling;RAC1 signaling pathway; Angiopoietin receptor Tie2-mediated signaling; EphrinB-EPHB pathway; Glypican 3 network; Syndecan-3-mediated signaling events; Ephrin B reverse signaling
**Proliferation**	No specific pathways specific

SM FIGURES

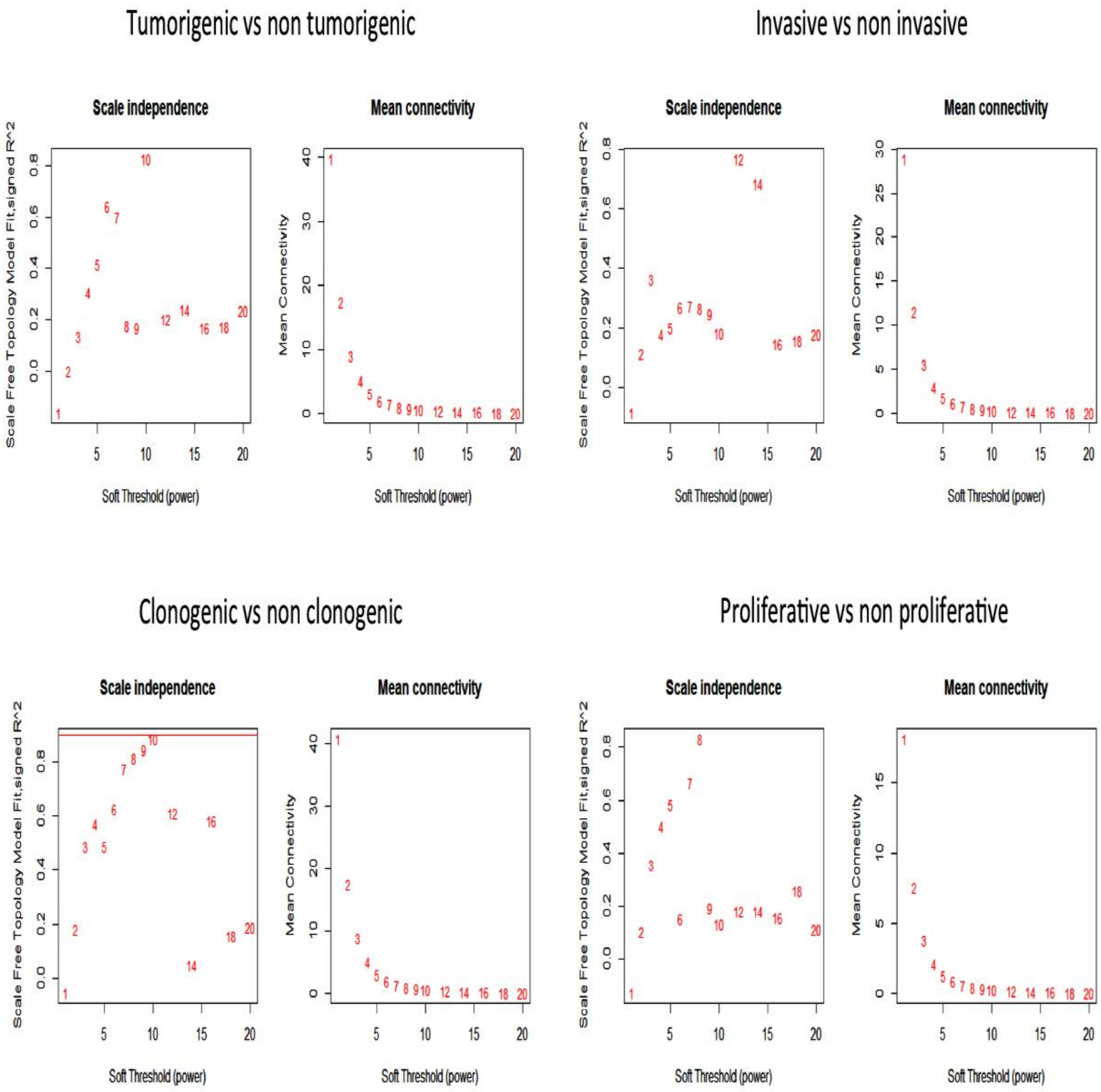

SM Fig. 1: Mean connectivity plot for gene co-expression network showing scale free property.

A) Tp (top-left), Ip (top-right), Cp (bottom-left), Pp (bottom-right)

B) **gene-gene co-expression networks**

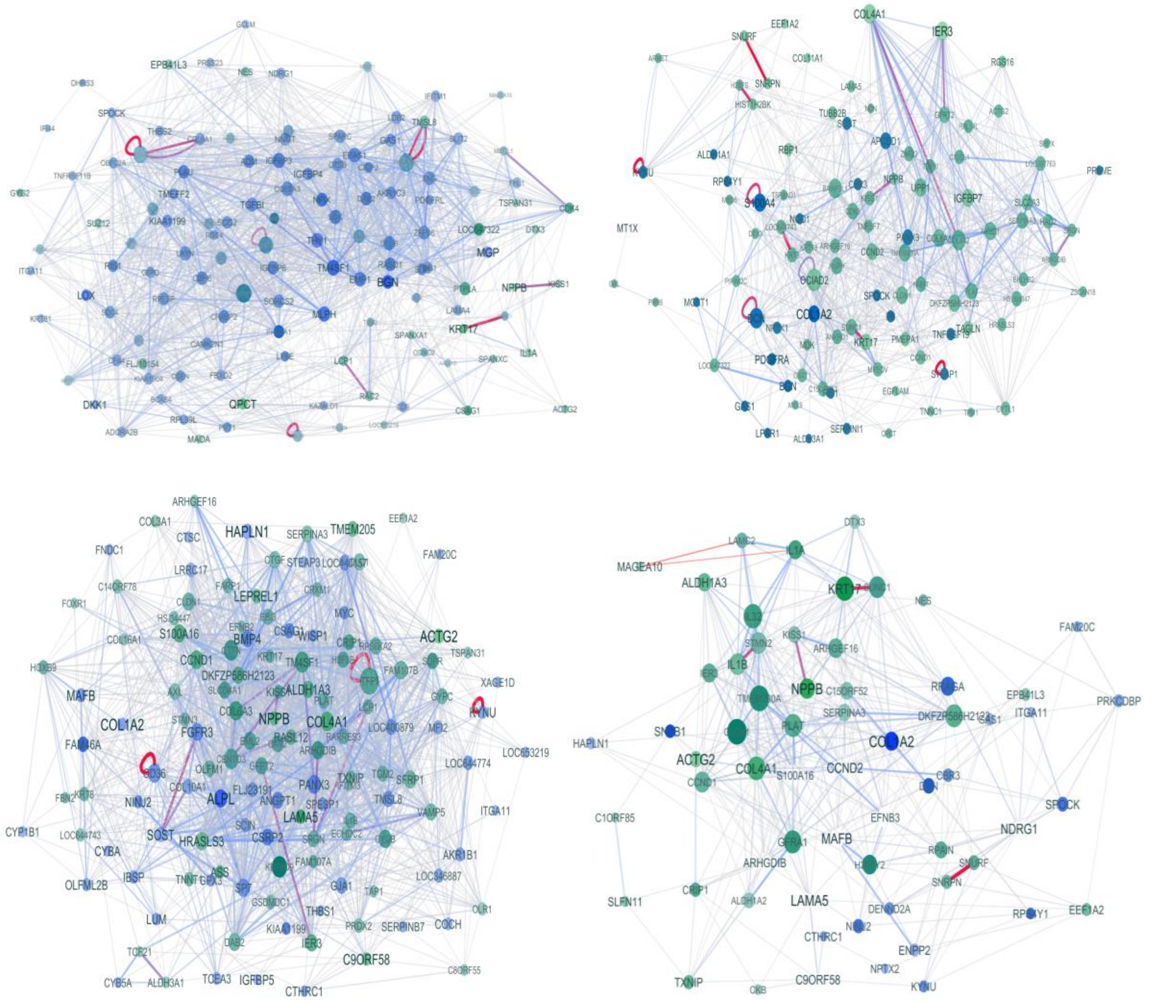

SM Fig. 2: **Gene – gene co-expression networks.** A) Tumorigenic, B) Invasive and C) Colony forming D) Proliferation phenotype. Bigger node size denotes more interactions with other nodes and colors from green to blue shows negative to positive differential expression, i.e. log(FC). The circles represent genes, and the bigger the more connected. The links connect genes weighted by co-expression of genes in the phenotypes**.** Tp, Cp and Ip present visible self-loops (self-regulating expression).

A) Core skeletons of DE miRNA-target gene networks

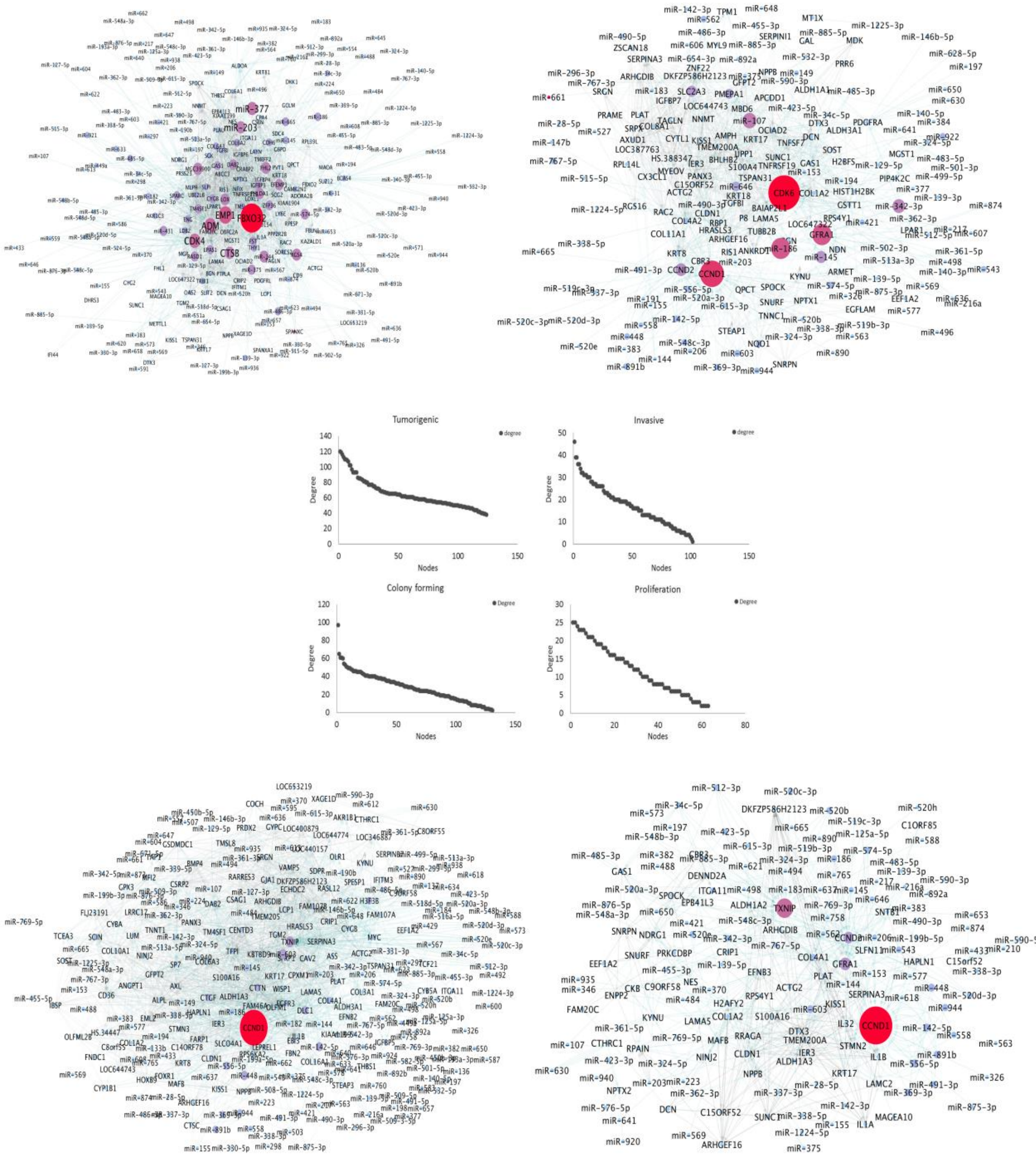

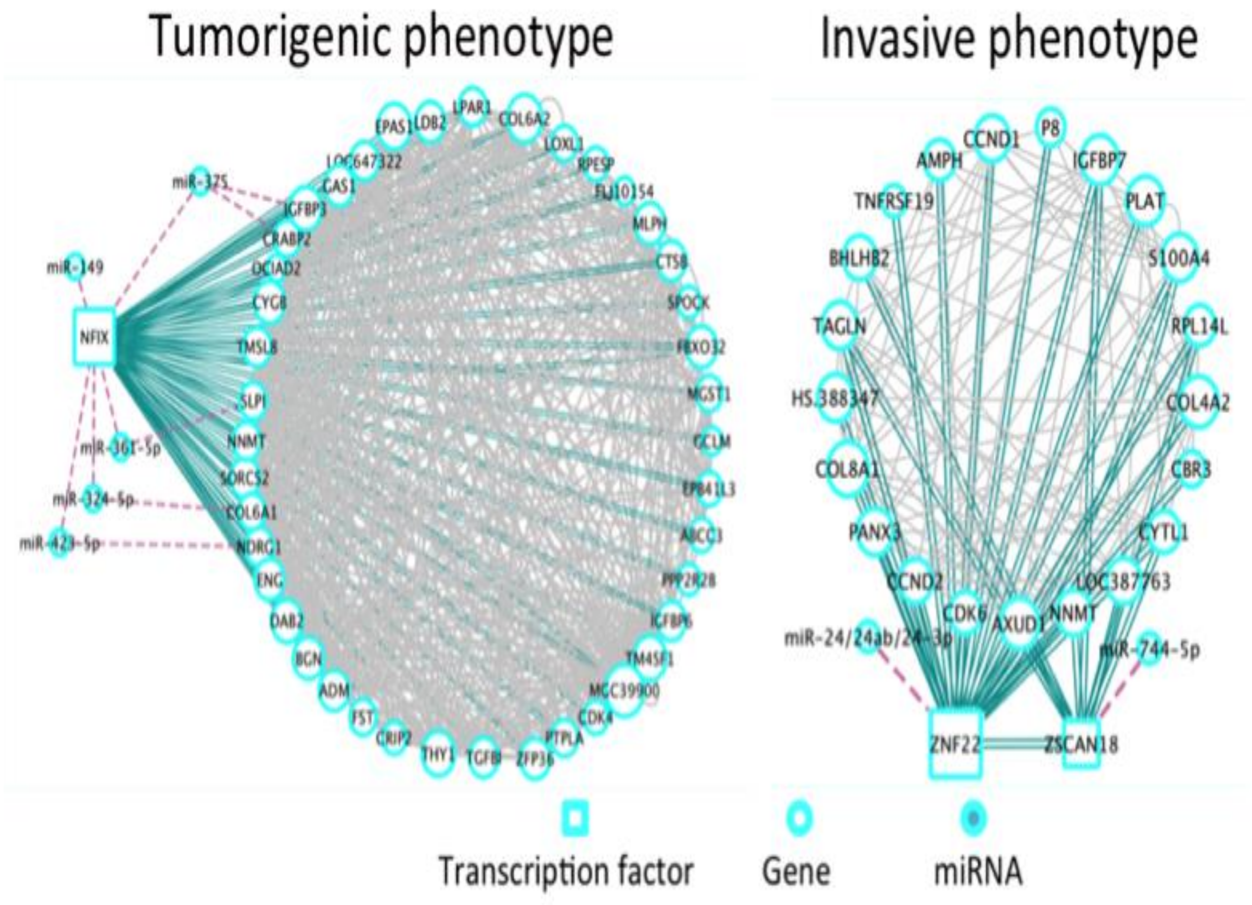

SM Fig. 3: Core skeletons of miRNA-target gene networks. Tp (top-left), Ip (top-right), Cp (bottom-left), Pp (bottom-right). Blue to Red colors and larger node size denote network cc. Higher DE hubs (large circles) characterize Tp - Ip, whereas non-hubs appear highly expressed in Cp-Pp. The DEGs in Cp and Pp networks show relatively higher network heterogeneity (Suppl Fig 13; Suppl file 2), and the higher characteristic path length indicates less efficiency in information transfer. Degree distribution of gene-gene co-expression networks shown in the middle.

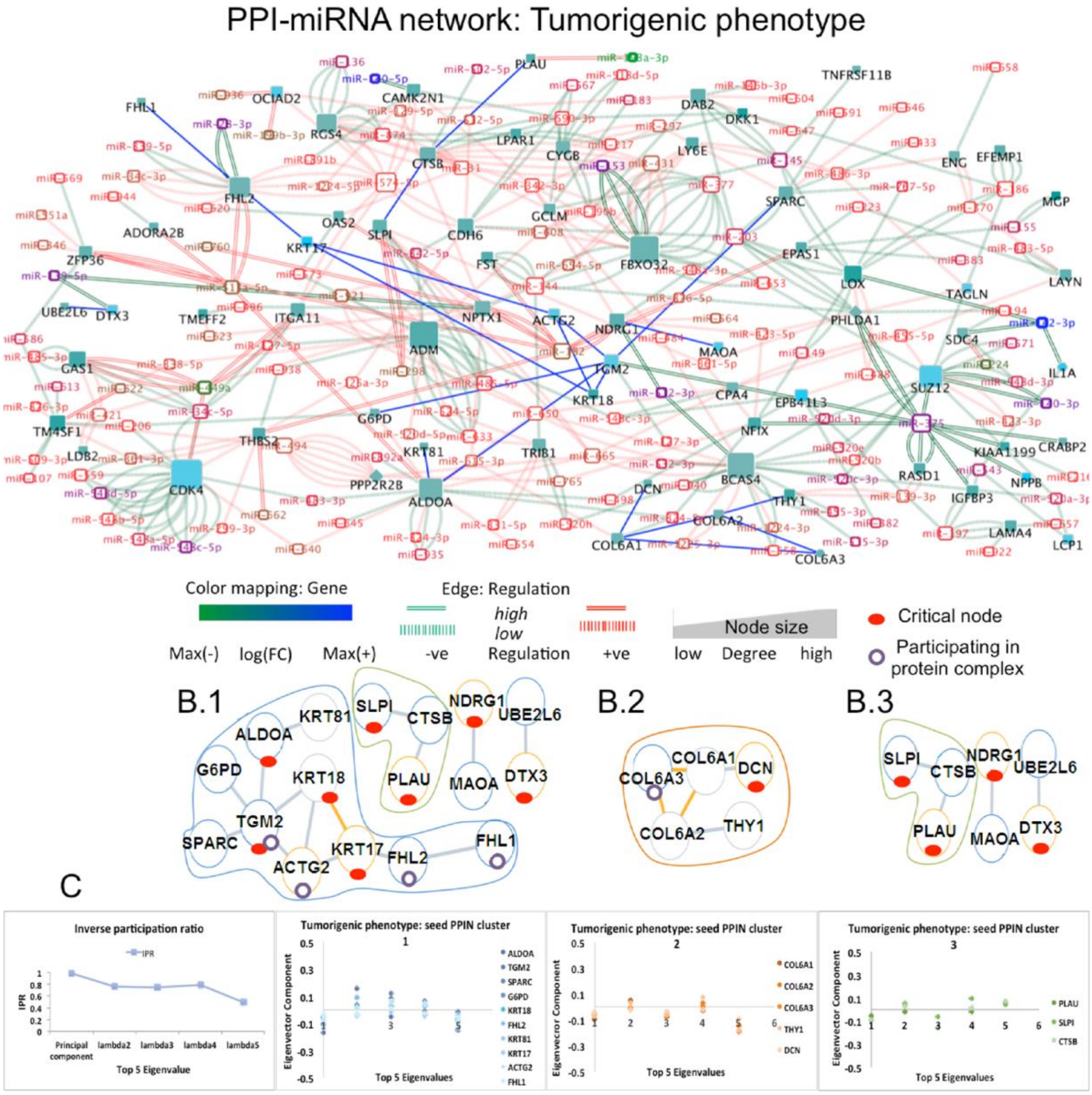

SM Fig. 4A. Tp PPI-miRNA network where edges originating high DE miRNAs are denoted by parallel lines and red color, while low expressions are denoted by dotted lines and grey color. Proteins involved in pathways appear with dashed lines and orange color, and protein-protein interactions with single blue line. Color and labels of the nodes are colored on the basis of the log(FC) of values for genes and miRNAs, respectively.

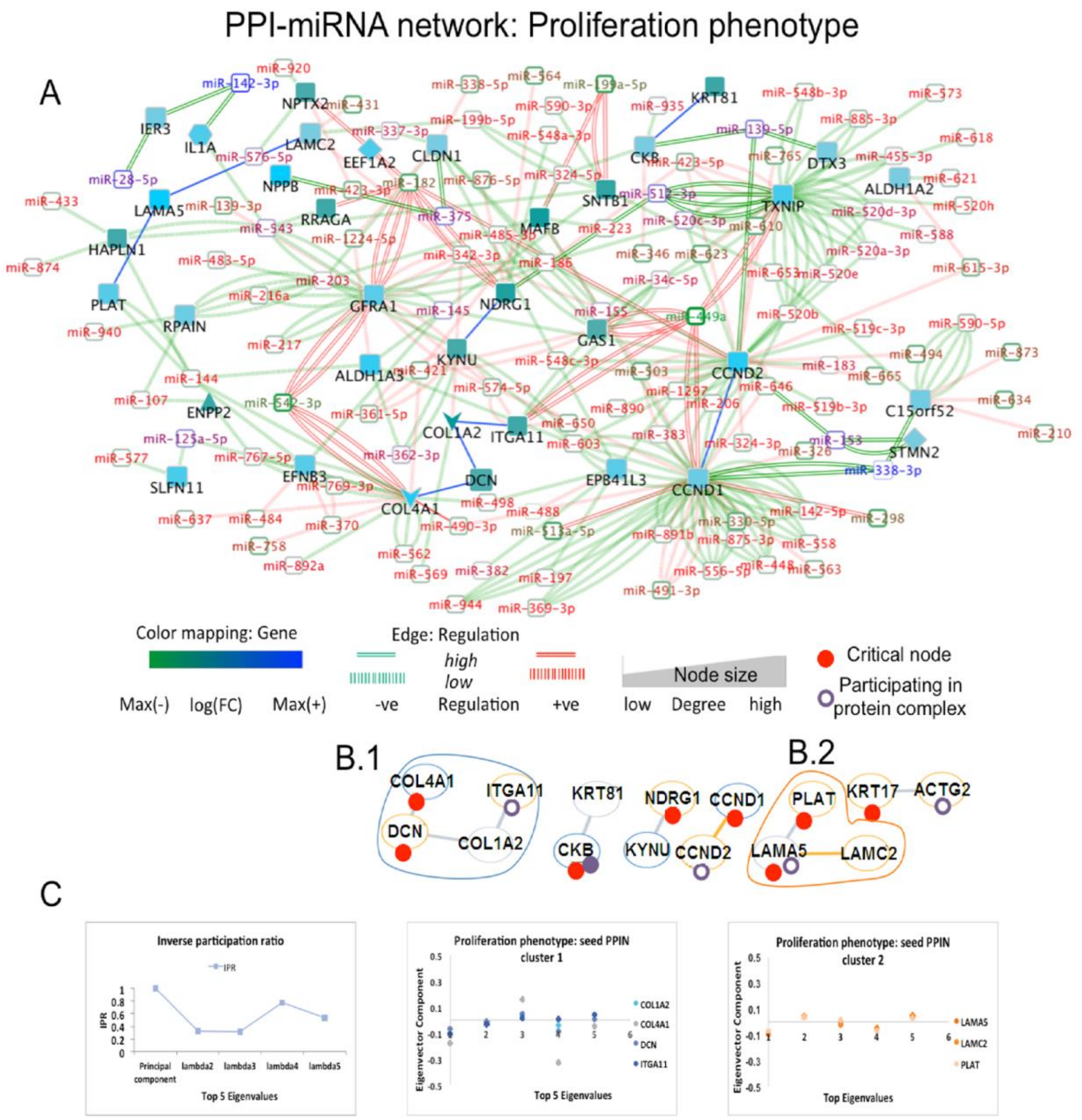

SM Fig. 4B. Pp PPI-miRNA network where edges originating high DE miRNAs are denoted by parallel lines and red color, while low expressions are denoted by dotted lines and grey color. Protein-protein interactions are shown with single blue line. Color and labels of the nodes are colored on the basis of the log(FC) values for genes and miRNAs, respectively.

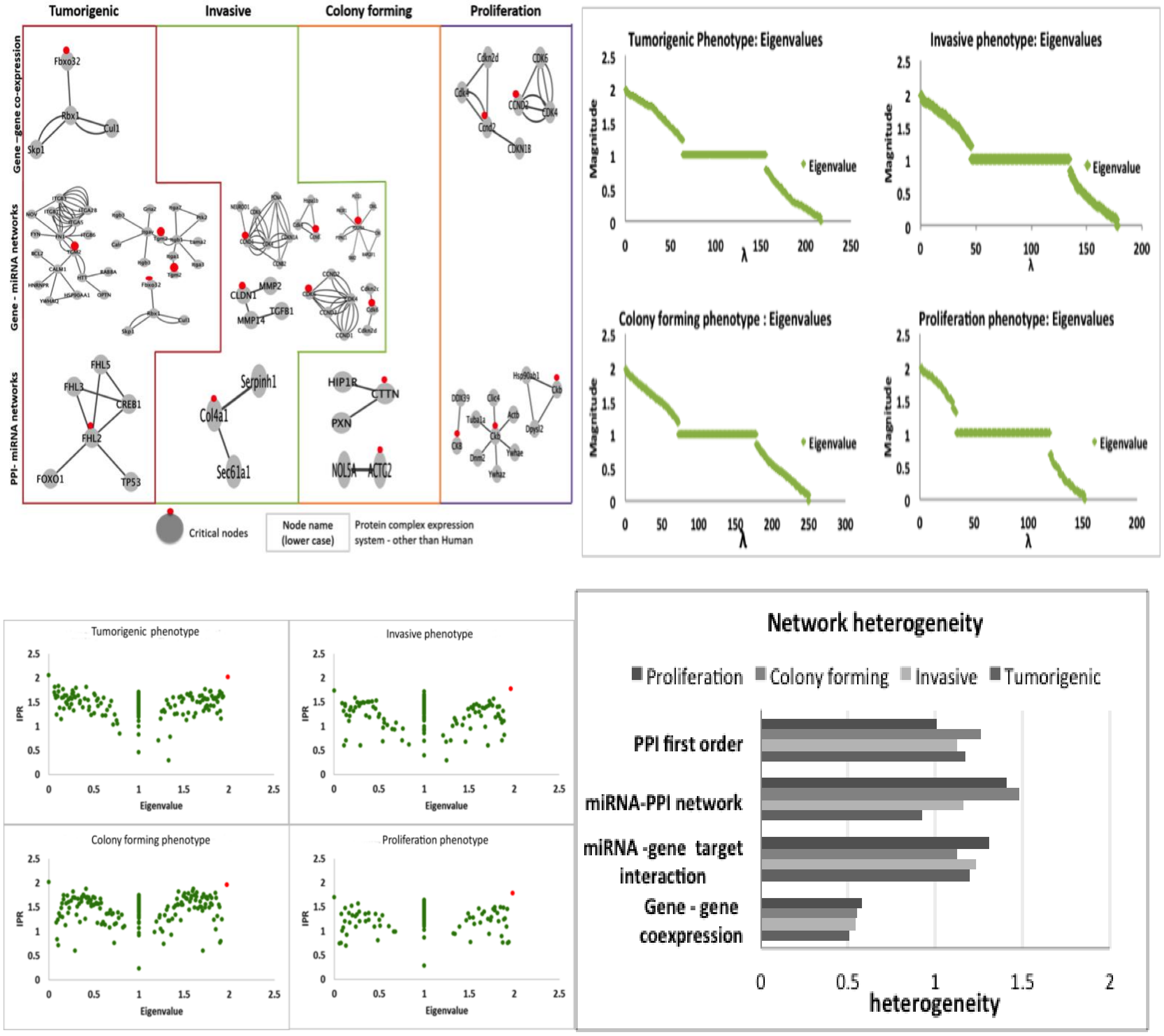

SM Fig. 5. Top-left**: Critical nodes in multitype networks showing participation in protein complexes.** The node names in upper case are experimentally verified in humans and node names in lower case are verified in other organism such as *Rattus Norvegicus* and *Mus Musculus*. Top-right: **Ranked eigenvalues** for PPIN-miRNA networks indicating typical trees-of-trees organization. Magnitude on Y-axis refers to minimized energy value (24). Bottom-left: **Inverse participation ratio (IPR)** vs Eigenvalues scatterplot showing similar trends as shown by (17) and illustrating that scale-free networks are best fitted with the measured data. The red dots are showing IPR for the principal Eigenvalue. Bottom-right: **Network heterogeneity**. It equals the coefficient of variation of the connectivity distribution, i.e. square root of variance of (K)/mean(K), where k is the connectivity of the node, which tells us about tendency of networks to contain hubs.

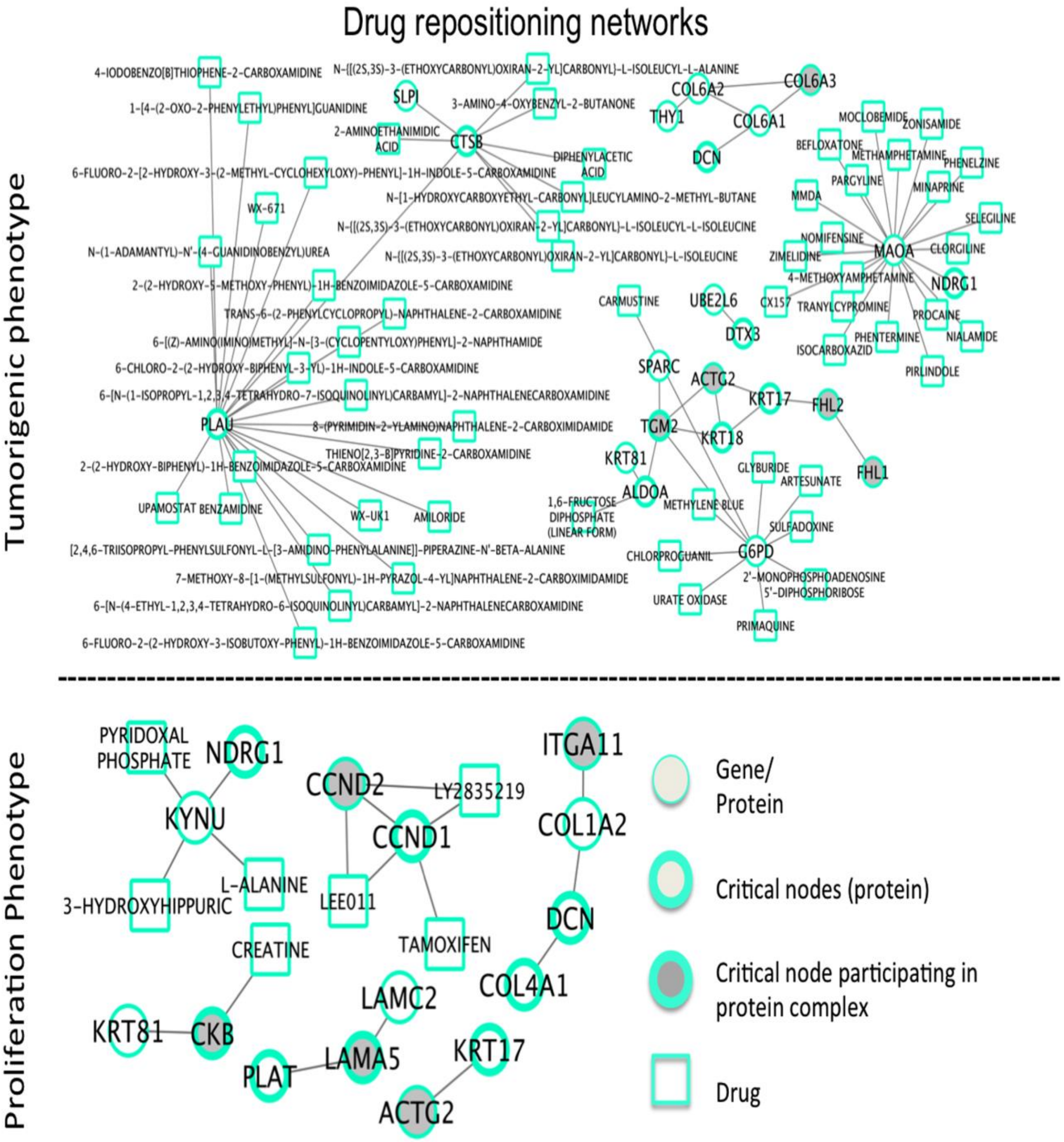

SM Fig. 6. **Drug repositioning networks.** For Tp and Pp drugs show experimentally verified interaction with genes.
